# Association between changes in cardiovascular health and the risk of multimorbidity: community-based cohort studies in the UK and Finland

**DOI:** 10.1016/j.lanepe.2024.100922

**Published:** 2024-05-06

**Authors:** Christof Prugger, Marie-Cécile Perier, Séverine Sabia, Aurore Fayosse, Thomas van Sloten, Xavier Jouven, Jaana Pentti, Mika Kivimäki, Jean-Philippe Empana

**Affiliations:** a Charité – Universitätsmedizin Berlin, Corporate Member of Freie Universität Berlin and Humboldt-Universität zu Berlin, Institute of Public Health, Seestraße 73, 13347, Berlin, Germany; b Université Paris Cité, Paris, INSERM U970, Paris Cardiovascular Research Centre (PARCC), 56 rue Leblanc, 75015, Paris, France; c Université Paris Cité, INSERM U1153, Epidemiology of Aging and Neurodegenerative Diseases, 10 avenue de Verdun, 75010, Paris, France; d Department of Epidemiology and Public Health, University College London, 1-19 Torrington Pl, London, Wc1E 7Hb, United Kingdom; e Department of Vascular Medicine, University Medical Centre Utrecht, Lundlaan 4, 3584 EA, Utrecht, the Netherlands; f Clinicum, Faculty of Medicine, University of Helsinki, Haartmaninkatu 8, 00290, Helsinki, Finland; g Department of Public Health, University of Turku, Kiinamyllynkatu 8-10, 20520, Turku, Finland; h Centre for Population Health Research, Turku University Hospital, University of Turku, Kiinamyllynkatu 8-10, 20520, Turku, Finland; i Finnish Institute of Occupational Health, Topeliuksenkatu 41 b, 00250, Helsinki, Finland; j UCL Brain Sciences, University College London, 17 Queen Square, WC1N 3AR, London, United Kingdom; k Clinicum, Faculty of Medicine, University of Helsinki, Tukholmankatu 8, 00290, Helsinki, Finland

**Keywords:** Cardiovascular health, Chronic diseases, Cohort study, Multimorbidity, Prevention

## Abstract

**Background:**

Better cardiovascular health is associated with lower risk of various chronic diseases, but its association with multimorbidity is poorly understood. We aimed to examine whether change in cardiovascular health is associated with multimorbidity risk.

**Methods:**

The primary analysis was conducted in the Whitehall II multiwave prospective cohort study (UK) and the validation analysis in the Finnish Public Sector cohort study (Finland). Change in cardiovascular health was assessed using the American Heart Association Life's Simple 7 (LS7) and Life's Essential 8 (LE8) at baseline and re-assessments, using objective measures in Whitehall II and self-reports and pharmacy claims in the Finnish Public Sector cohort study, respectively. Multimorbidity was defined as the presence of two or more of 12 chronic diseases during follow-up. We estimated hazard ratios (HR) and 95% confidence intervals (CI) using Cox's proportional hazard models with age as time scale, adjusting for sex, education, occupation, marital status, and ethnicity.

**Findings:**

In the primary analysis among 9715 participants, mean age was 44.8 (standard deviation 6.0) years and 67.6% participants were men at baseline. During the median follow-up of 31.4 (interquartile range 26.8–32.3) years, 2751 participants developed multimorbidity. The hazard of multimorbidity decreased by 8% (HR 0.92, 95% CI 0.88–0.96) per ideal LS7 metric increment over 5 years and by 14% (HR 0.86, 95% CI 0.80–0.93) per ten points increase in LE8 score over 10 years. These findings were replicated in the validation analysis among 75,377 participants in terms of 4-year change in cardiovascular health.

**Interpretation:**

Improvement in cardiovascular health was associated with lower multimorbidity risk in two community-based cohort studies. Interventions improving cardiovascular health of the community may contribute to multimorbidity prevention.

**Funding:**

None.

**Research in context**

**Evidence before this study**

The concept of primordial prevention, introduced in 1978, gained renewed attention in 2010 when the American Heart Association defined ‘ideal cardiovascular health.’ This concept refers to healthy behaviours (not smoking, engaging in regular physical activity, maintaining a healthy diet, managing weight, getting adequate sleep) and optimal levels of biological factors (blood pressure, fasting blood glucose, and non-HDL cholesterol). It is measured using the Life's Simple 7 (LS7) and Life's Essential 8 (LE8) scores, with sleep included only in LE8. We searched PubMed, Embase, Google Scholar, and Web of Science for articles published in English or French from February 2010 to October 17, 2023, using the following search terms: ‘cardiovascular health’, ‘Life's Simple 7′, ‘Life's Essential 8′ with ‘cardiovascular disease’, ‘coronary heart disease’, ‘stroke’, ‘heart failure’, ‘arrhythmic events’, ‘venous thromboembolism’, ‘peripheral artery disease’, ‘type 2 diabetes’, ‘depression’, ‘arthritis’, ‘cancer’, ‘renal disease’, ‘dementia’, ‘chronic obstructive pulmonary disease’, ‘thyroid disease’, and ‘multimorbidity’. We found 295 studies that reported LS7-measured ideal cardiovascular health to be associated with reduced risk of cardiovascular disease and its various presentations, 19 studies on decreased risk of dementia, cognitive decline, and cognitive impairment, 11 studies on reduced type 2 diabetes risk (including one systematic review), 16 studies on reduced depression (including one systematic review), one on arthritis, seven on cancer, and two on renal diseases. Similar associations were observed when cardiovascular health was assessed using LE8. One prospective study, the UK Biobank, reported an association between ideal cardiovascular health and a reduced risk of multimorbidity. However, no longitudinal studies were found that investigated the relationship between changes in cardiovascular health over time and subsequent multimorbidity risk.

**Added value of this study**

To our knowledge, this is the first longitudinal study exploring the association between change in cardiovascular health over time and incident multimorbidity. Our definition of multimorbidity covered a range of conditions, including coronary heart disease, stroke, heart failure, chronic obstructive pulmonary disease, chronic kidney disease, liver disease, depression, dementia, other mental disorders, Parkinson's disease, arthritis/rheumatoid arthritis, and cancer. In the primary analysis of the Whitehall II multi-wave cohort study in the UK, we found that each incremental improvement in the number of ideal LS7 metrics (ranging from 0 to 7) between baseline and a 5-year re-assessment was associated with an 8% decrease in the subsequent risk of developing multimorbidity over a 26.5-year follow-up period. Additionally, a 10-point increase in the LE8 score (ranging from 0 to 100) over 10 years was associated with a 14% reduction in the risk of multimorbidity. The findings were similar in validation analyses conducted on an independent community-based prospective study from Finland. Both in the Whitehall II and Finnish study populations, achieving ≥2 ideal LS7 metrics was estimated to prevent 53% of multimorbidity cases among individuals initially lacking any LS7 metrics.

**Implications of all the available evidence**

The ageing of the population, combined with the obesity epidemics, has made multimorbidity a significant societal and public health concern. Globally, multimorbidity affects 37% of adults and more than half of adults over 60 years old. These numbers are projected to rise, calling for immediate and effective preventive strategies. Our results are consistent with previous studies demonstrating robust associations between ideal cardiovascular health and reduced risks of both individual diseases and multimorbidity. Furthermore, we show that individuals who improved their cardiovascular health over 5 and 10 years experienced a significantly reduced risk of developing multimorbidity. Collectively, this evidence suggests that primordial prevention targeting factors of the American Heart Association's Life's Simple 7 and Life's Essential 8 scores may represent an effective strategy for alleviating the burden of multimorbidity in the community.

## Introduction

To guide individuals in adopting habits that lower risk of developing heart disease or stroke, the American Heart Association (AHA) has launched the concept of ‘cardiovascular health’ (CVH), referring to healthy behavioural (not smoking, engaging in regular physical activity, maintaining a healthy diet, managing weight, getting adequate sleep) and optimal levels of biological factors (blood pressure, fasting blood glucose, and non-HDL cholesterol).^1^^,^^2^ Supporting this, observational studies have shown that better CVH assessed by scores like Life's Simple 7 (LS7) or Life's Essential 8 (LE8) is associated with reduced risk of coronary heart disease, stroke, and heart failure.^3^, ^4^, ^5^, ^6^ In addition, there is evidence that people with better CVH also have lower risks of non-cardiovascular conditions, such as cancer,^7^, ^8^, ^9^ dementia,^10^^,^^11^ depression,^12^ diabetes,^13^ liver disease,^14^ and chronic kidney disease.^15^ Yet, CVH's relationship with multimorbidity (presence of two or more chronic conditions) remains unclear.

The prevalence of multimorbidity has risen in recent decades, currently affecting 37% of adults globally and more than half of adults worldwide over 60 years.^16^ With population ageing, these figures are projected to rise, calling for immediate interventions.^16^ Employing a unified strategy that targets several health behaviours and factors, like the CVH components, may hold potential for synergistic prevention of diverse morbidities. However, the only prior study that prospectively examined CVH in relation to multiple chronic diseases was limited to four conditions, i.e., cardiovascular disease, diabetes, cancer, and dementia.^17^ Furthermore, no prior study has investigated the association of change in CVH with multimorbidity risk, whereas change in CVH has been related to single cardiovascular disease and non-cardiovascular outcomes.^4^^,^^7^^,^^12^^,^^13^

We aimed to investigate whether better CVH and its improvement are associated with lower multimorbidity risk considering 12 chronic diseases of considerable public health significance. To examine associations of CVH and multimorbidity risk in different contexts, we used clinical measurements of CVH factors in the primary analysis and considered CVH factors based on self-reports and pharmacy claims in the validation analysis.

## Methods

This study was approved by the University College London Hospital Committee on the Ethics of Human Research (reference number 85/0938) and the Helsinki Uusimaa Hospital District Ethics Committee (60/13/03/00/11). Participants provided written informed consent at each contact. A data access application summarizing our study plan was submitted before conducting the study. This summary is added to the Appendix. Our study follows the STROBE reporting guidelines for cohort studies. Please see the filled in STROBE checklist in the Appendix.

### Study design and populations

The primary analysis was conducted using data from the Whitehall II (WHII) study among British civil servants, while the validation analysis used data from the Finnish Public Sector (FPS) study among Finnish public employees.^18^^,^^19^ WHII is a closed cohort of 10,308 participants at baseline (1985–1988) with re-assessments every 4 or 5 years. The target population was all men and women aged 35–55 working in the London offices of 20 civil-service departments.^18^ Eligible people were invited to participate by letter. The response rate after excluding those who were ineligible was 73% (74% among men, 71% among women). The protocol of the most recent study phase is provided in the Appendix. FPS is an open cohort of participants aged 17–77 years recruited in multiple surveys with re-assessments every 4 years. The target population comprised public sector personnel from 10 cities and 21 hospitals within the same geographical areas at the time of data collection.^19^ This included individuals eligible for at least one of the four surveys conducted in 2000–2002, 2004–2005, 2008–2009, and 2012–2013, provided they had a job contract of at least 6 months before the survey. The overall response rate was 68%, with response rates varying between 67% and 69% in each survey.

In WHII, we used data from clinical examinations and questionnaires collected at baseline, in 1991–1993, and 1997–1999 and a follow-up of multimorbidity until 31 March 2019 (Supplementary Figure S1 in the Appendix). In FPS, we used data from questionnaires and pharmacy claims at baseline (2000–2002, 2004–2005, 2008–2009) and re-assessments (2004–2005, 2008–2009, 2012–2013) and a follow-up of multimorbidity until 31 December 2016 (Supplementary Figure S2 in the Appendix).

We included in this study all WHII and FPS participants without history of cardiovascular disease and multimorbidity, and with complete data on CVH and covariates at the start of multimorbidity follow-up. The follow-up started at baseline for the analysis of CVH level and after re-assessment for the analysis of change in CVH.

### Measurements

In the WHII study, smoking status, physical activity, and diet were assessed using questionnaire. Body mass index, blood pressure, fasting glucose, and blood cholesterol were collected using standard protocols. Weight was measured in underwear to the nearest 0.1 kg on Soehnle electronic scales with digital readout (Leifheit, Nassau, Germany), and height was measured in bare feet to the nearest 1 mm using a stadiometer with the participant standing erect with the head in the Frankfurt plane. Systolic and diastolic blood pressures were measured twice in the sitting position after 5 min of rest with the Hawksley random-0 sphygmomanometer. The average of each of the systolic and diastolic blood pressure readings was used in the analysis. Venous blood samples were taken after at least 5 h of fasting, and serum obtained after centrifugation was refrigerated at 4 °C and assayed within 72 h of the blood draw. Total cholesterol was measured using a Cobas Fara centrifugal analyzer (Roche Diagnostics, Nutley, NJ). Sleep duration was assessed by questionnaire using the question ‘how many hours of sleep do you have on an average week-night?” Response categories were 5 h or less, 6 h, 7 h, 8 h, and 9 h or more.

As in WHII, smoking, physical activity and sleep duration were measured using questionnaire in the FPS study. In addition, weight and height were self-reported, while the assessment of blood pressure, fasting blood glucose, and total cholesterol was based on linked records on treatments (antihypertensives, the Anatomical Therapeutic Chemical (ATC) codes C02,C03,C07,C08,C09; antidiabetic medication, ATC A10; statins, ATC C10AA) from the Drug Reimbursement Register of the Social Insurance Institution of Finland and diagnoses (hypertension, ICD-10 I10–I15; diabetes, ICD-10 E10-E14) from the National Hospital Discharge Register kept by the Finnish Institute for Health and Welfare.

### Assessment of cardiovascular health

In WHII, CVH was assessed by the LS7 (considering smoking, physical activity, diet, body mass index, blood pressure, fasting blood glucose, and total cholesterol) and LE8 metrics (additionally including sleep duration, providing more granularity in the scoring of each metric, and offering the possibility to consider nicotine exposure, non-HDL cholesterol and HbA1C) according to AHA criteria,^1^^,^^2^ as detailed in Supplementary Tables S1 and S2 in the Appendix. Briefly, each LS7 metric is weighted by 0, 1, or 2 to reflect poor, intermediate and ideal level. This permits to consider the number of ideal LS7 metrics (range 0–7), the LS7 score (range 0–14 points), and poor, intermediate, and high CVH defined by 0–2, 3–4, and 5–7 ideal metrics or 0–5, 6–9, and 10–14 points, respectively.^4^^,^^5^ Briefly, ideal metrics are no smoking, moderate (at least 150 min per week) or vigorous (at least 75 min per week) physical activity, optimal dietary items, body mass index <25 kg/m^2^, untreated blood pressure <120/80 mmHg, untreated fasting blood glucose <100 mg/dL (<5.6 mmol/l), and untreated total cholesterol <200 mg/dL (<11.1 mmol/l). Each LE8 metric is scored from 0 to 100 and their sum is averaged to derive the LE8 score (range 0–100 points), from which low (0–49), moderate (50–79), and high (80–100) CVH was defined.^2^

In FPS, only diagnoses and treatments were available for blood pressure, fasting blood glucose, and total cholesterol, precluding to assess LS7 and LE8 metrics according to AHA criteria. Further, data on healthy diet were not available. Each LS7 metric was therefore categorized as ideal vs. non-ideal as detailed in Supplementary Table S3 in the Appendix, with the number of ideal metrics possibly ranging from 0 to 6, and poor, intermediate, and high CVH defined by 0–2, 3–4, and 5–6 ideal metrics. To resemble the LE8 score, we additionally considered ideal LS7 metrics plus sleep duration (LS7^+^, possible range 0–7), defining ideal level of sleep duration as 6 to ≤9 h as in the LE8.

### Exposures: baseline and change in cardiovascular health

Primary exposures in WHII were baseline and 5-year change (from baseline to 1991–1993) in the number of ideal LS7 metrics and baseline and 10-year change (from baseline to 1997–1999) in LE8 score. In FPS, there were baseline and 4-year change (from baseline to re-assessment) in the number of ideal LS7 metrics.

Secondary exposures were baseline level and 5-year change in LS7 score in WHII and baseline and 4-year change in the number of ideal LS7^+^ in FPS.

We first examined changes in LS7 and LE8 as continuous exposures. For WHII, this involved assessing the absolute differences in LS7 between study baseline and 5-year re-assessment (re-assessment value—baseline value), including the difference in the number of LS7 ideal metrics between the two time points in the main analysis, and the difference in the LS7 score between the two time points in the secondary analysis. In addition, we examined the absolute difference in LE8 score between the 10-year re-assessment and baseline.

We then examined categorical changes in LS7 and LE8 during the same time intervals. For this, we cross-tabulated the categories of LS7 (based on the number of ideal metrics in the main analysis and the level of LS7 score in the secondary analysis) at baseline and the 5-year re-assessment, and the categories of LE8 at baseline and the 10-year re-assessment. Some categories of change were either underrepresented or contained few (<5) or no multimorbidity events. To obtain sufficient numbers for each category, we grouped together those who changed ‘from poor to intermediate’ and ‘from poor to high’ LS7, those who changed ‘from high to moderate’ and ‘from high to low’ LE8, and those who changed ‘from low to moderate’ and ‘from low to high’ LE8 categories.

In the validation analysis based on FPS, we used the same approach and the same merged LS7 categories to examine 4-year change in cardiovascular health.

### Outcomes: multimorbidity

The primary outcome in both cohorts was multimorbidity status, defined as the presence of two or more of 12 chronic diseases during follow-up, ascertained through record linkage with UK National Health Services (NHS) data, i.e., the Hospital Episodes Statistics (HES) in WHII^20^, ^21^, ^22^ and by linkage with the National Hospital Discharge Registers (HILMO), the National Drug Reimbursement Register, and the National Purchase Register in FPS. The 12 chronic diseases were: coronary heart disease, stroke, heart failure, chronic obstructive pulmonary disease, chronic kidney disease, liver disease, depression, dementia, other mental disorders, Parkinson disease, arthritis/rheumatoid arthritis, and cancer. Diabetes was not considered, because glycaemic status is included in the LS7 and LE8. Criteria used to define the chronic diseases are given in Supplementary Table S4 in the Appendix. The secondary outcome in both cohorts was multimorbidity severity, defined as the total number of the 12 diseases accumulated during follow-up, considering 2 (least severe), 3, and 4 or more conditions (most severe).

### Covariates

Baseline covariates were sex (women, men), education (WHII: lower secondary school or less, higher secondary school, university or higher degree, unknown; FPS: primary, secondary, tertiary or higher), occupation (WHII: administrative, professional/executive, clerical/support; FPS: low, intermediate, high), marital status (WHII: married/cohabiting, single, divorced, widowed; FPS: spouse, no spouse), and ethnicity (WHII: white, non-white [combination of South Asian, Black, other] as self-reported; not available in FPS).

### Statistical analysis

After checking the proportionality assumption using graphical diagnostics based on Schoenfeld residuals, we estimated hazard ratios (HR) with 95% confidence intervals (CI) for associations of each exposure of interest with multimorbidity status during follow-up using Cox's proportional hazards models with age as time scale and birth cohort strata (5- and 10-year strata in WHII and FPS, respectively), adjusting for the above-mentioned baseline covariates. Models with the exposure variables per one ideal LS7/LS7^+^ metric increase, per one-point LS7 score increase, and per 10 points LE8 score increase over time were additionally adjusted for baseline LS7/LE8 values. When examining CVH score as a continuous exposure, the linearity assumption was assessed by comparing the likelihood ratio of models with and without quadratic term on the CVH score. No major departure from linearity was observed in both cohorts. Data were censored at date of multimorbidity diagnosis, death to account for competing risk,^23^ or end of follow-up, whichever came first.

In main analyses, we estimated associations of baseline CVH with incident multimorbidity status using categories of the number of ideal LS7 metrics (0–1 as reference) and per one ideal LS7 metric in WHII and FPS and using categories of LE8 score (low level as reference) and per 10 points LE8 score in WHII. Regarding the CVH change analysis, we drew cumulative incidence curves for incident multimorbidity status by categories of change in ideal LS7 metrics in WHII and in FPS. We then estimated associations of change in CVH with multimorbidity status using categories of change in ideal LS7 metrics (persistently low level as reference) and per one ideal LS7 metric increase in WHII and FPS as well as using categories of change in LE8 score (persistently low level as reference) and per 10-point LE8 score increase in WHII.

In secondary analyses, we calculated population preventable fractions (PPF) for achieving ≥2 ideal LS7 metrics based on the corresponding HR and prevalence.^24^ We estimated odds ratios (OR) with 95% CI of multimorbidity severity per one ideal LS7 metric at baseline and per one ideal LS7 metric increase from baseline to re-assessment in WHII and FPS, using multinomial logistic regression models adjusted for the above-mentioned baseline covariates and considering participants with 2, 3, and 4 or more conditions as compared to those with 0–1 condition. Further, we estimated associations of baseline LS7 score and its 5-year change and of baseline ideal LS7^+^ metrics and its 4-year change with multimorbidity status in WHII and FPS, respectively. We fitted Cox's proportional hazards models for each individual LS7 metric and sleep duration at baseline (non-ideal as reference) and its change between baseline and re-assessment (persistently non-ideal as reference) in WHII and FPS.

We conducted several sensitivity analyses to investigate the robustness of our findings in WHII. First, we used inverse probability weighting to account for possible non-random cohort attrition in the change analysis.^25^ Weights corresponded to the inverse probabilities of attending the clinical examination in 1991–1993/1997–1999 and having complete data on LS7/LE8 (separate analyses) estimated in multivariate logistic regression analysis using baseline characteristics. Second, we used Fine and Gray sub-distribution hazard models to account for deaths as competing events.^26^ Third, we repeated the analysis in subcohorts of participants who were free of any of the 12 chronic diseases at baseline and re-assessment to ensure CVH status is unaffected by any first condition. Fourth, we limited follow-up to 15 years to resemble the follow-up time of FPS participants. Fifth, we excluded coronary heart disease, stroke, and heart failure from multimorbidity to examine whether cardiovascular disease drove the observed associations. Lastly, we investigated residual confounding additionally adjusting for post-hoc variables including family history of single diseases (angina, myocardial infarction, stroke, diabetes, cancer death), area-based deprivation (Townsend score of census ward),^27^ and alcohol consumption.

The statistical analyses were conducted using SAS Version 9.4 (Cary, USA) and R version 4.2.1 (Vienna, Austria). Syntax for the analyses is provided in the Appendix.

### Role of the funding source

The funders of the study had no role in study design, data collection, data analysis, data interpretation, or writing of the report. All authors had final responsibility for the decision to submit for publication.

## Results

Table 1 presents characteristics of the study populations at baseline by categories of number of ideal LS7 metrics in 9715 WHII participants and 75,377 FPS participants (flow charts in Supplementary Figures S3 and S4 in the Appendix). Overall, mean age (standard deviation) was 44.8 (6.0) and 43.3 (SD 10.1) years, and 6567 (67.6%) were men while 60,221 (79.9%) were women in WHII and FPS, respectively. In WHII, 2206 participants (22.7%) had 5-7 ideal LS7 metrics, and 1594 participants (16.4%) had 0-2 ideal LS7 metrics. In FPS, 44,510 participants (59.0%) had 5-6 ideal LS7 metrics, compared to 2469 participants (3.3%) who had 0-2 ideal LS7 metrics. By design, there were no prevalent CHD or stroke at baseline. In WHII, only 113 (1.2%) and 73 (0.8%) participants had depression and cancer, and in FPS, 9100 (12.1%) and 733 (1.0%) participants had these conditions at baseline. Additional conditions solely available in FPS were even less prevalent ranging from 4 (0.01%) participants having dementia to 1091 (1.45%) having arthritis at baseline. Baseline characteristics by categories of LE8 score points (in 9729 WHII participants) and by number of ideal LS7^+^ metrics (in 74,926 FPS participants) are shown in Supplementary Table S5 in the Appendix.

**Table 1:** Baseline characteristics of the Whitehall II study and Finnish Public Sector study populations by categories of number of ideal LS7 metrics.

Whitehall II study (N = 9715)				Finnish Public Sector study (N = 75,377)^a^			
	Poor (0–2)	Intermediate (3–4)	High (5–7)		Poor (0–2)	Intermediate (3–4)	High (5–6)
	N = 1594	N = 5915	N = 2206		N = 2469	N = 28,398	N = 44,510
Age, mean (SD), y	46.5 (6.0)	45.1 (6.0)	43.0 (5.6)	Age, mean (SD), y	51.1 (8.2)	45.4 (9.7)	41.6 (9.9)
Men, n (%)	981 (61.5)	4082 (69.0)	1504 (68.2)	Men, n (%)	789 (32.0)	6948 (24.5)	7419 (16.7)
Education level, n (%)				Education level, n (%)			
Lower secondary school or less	523 (32.8)	1536 (26.0)	443 (20.1)	Primary	495 (20.1)	3648 (12.9)	2654 (6.0)
Higher secondary school	298 (18.7)	1081 (18.3)	422 (19.1)	Secondary	952 (38.6)	10,990 (38.7)	13,163 (29.6)
University or higher degree	389 (24.4)	1807 (30.6)	821 (37.2)	Tertiary or higher	1022 (41.4)	13,760 (48.5)	28,693 (64.5)
Unknown	384 (24.1)	1491 (25.2)	520 (23.6)				
Occupation, n (%)				Occupation, n (%)			
Administrative	347 (21.8)	1793 (30.3)	758 (34.3)	Low	1125 (45.6)	12,054 (42.5)	13,627 (30.6)
Professional/executive	692 (43.4)	2906 (49.1)	1123 (50.9)	Intermediate	771 (31.2)	9142 (32.2)	15,355 (34.5)
Clerical/support	555 (34.8)	1216 (20.6)	325 (14.7)	High	573 (23.2)	7202 (25.4)	15,528 (34.9)
Marital status, n (%)				Marital status, n (%)			
Married/cohabiting	1099 (68.9)	4437 (75.0)	1670 (75.7)	Spouse	1836 (74.4)	21,475 (75.6)	33,717 (75.8)
Single	297 (18.6)	938 (15.9)	356 (16.1)	No spouse	633 (25.6)	6923 (24.4)	10,793 (24.3)
Divorced	169 (10.6)	463 (7.8)	162 (7.3)				
Widowed	29 (1.8)	77 (1.3)	18 (0.8)				
Ethnicity, white, n (%)	1376 (86.3)	5366 (90.7)	2039 (92.4)	Ethnicity	NA	NA	NA

LS7, Life's Simple 7; NA, not available; SD, standard deviation.

a The diet metric was unavailable in the Finnish Public Sector study, explaining why the number of ideal metrics ranges from 0 to 6.

During a median (interquartile range [IQR]) follow-up of 31.4 (26.8–32.3) and 14.7 (12.2–16.2) years from baseline, 2751 and 6475 multimorbidity events occurred, corresponding to incidence rates (95% CI) of 10.0 (9.6–10.3) and 6.4 (6.3–6.6) per 1000 person-years in WHII and FPS, respectively. The most frequent combinations of two conditions were coronary heart disease and cancer in WHII and depression and arthritis in FPS (Supplementary Table S6 in the Appendix). Most frequent combinations of three conditions are in Supplementary Table S7 in the Appendix. These Supplementary Tables S6 and S7 also show multimorbidity combinations in case we had included diabetes.

As shown in Table 2, in WHII, the adjusted HR (95% CI) of multimorbidity decreased from 0.60 (0.50–0.73) for two ideal LS7 metrics to 0.30 (0.22–0.40) for 6–7 ideal LS7 metrics as compared to 0–1 ideal LS7 metric. The hazard of multimorbidity decreased by 21% (HR 0.79, 95% CI 0.76–0.82) per ideal LS7 metric. In FPS, the analysis largely confirmed these results.

**Table 2:** Associations of ideal LS7 metrics at baseline with multimorbidity status during follow-up in the Whitehall II study and Finnish Public Sector study populations.

	Whitehall II study				Finnish Public Sector study			
	n MM/N	IR per 1000 PY	HR	95% CI	n MM/N	IR per 1000 PY	HR	95% CI
No. of ideal LS7 metrics								
0–1	130/244	21.9	Ref		102/439	20.2	Ref	
2	522/1350	14.6	0.60	0.50–0.73	387/2030	15.2	0.79	0.63–0.98
3	953/2954	11.5	0.48	0.40–0.58	1284/8665	11.3	0.66	0.54–0.81
4	745/2961	8.7	0.39	0.32–0.47	2022/19,733	7.7	0.50	0.41–0.61
5	325/1737	6.3	0.30	0.25–0.37	1830/26,203	5.2	0.38	0.31–0.46
6–7^a^	76/469	5.4	0.30	0.22–0.40	850/18,307	3.5	0.28	0.23–0.35
Per one ideal LS7 metric	2751/9715		0.79	0.76–0.82	6475/75,377		0.77	0.75–0.79

CI, confidence interval; HR, hazard ratio; IR, incidence rate; LS7, Life's Simple 7; MM, multimorbidity; OR, Odds ratio; PY, person-years.

Baseline examinations took place between 1985 and 1988 in the Whitehall II study and in 2000–2002, 2004–2005, and 2008–2009 in the Finnish Public Sector study (open cohort).

Hazard ratios and 95% confidence intervals from Cox's proportional hazards models with age as time scale and birth cohort strata, adjusted for sex, education, occupation, and marital status in Finnish Public Sector study and additionally for ethnicity in Whitehall II study.

a Maximum number of 6 ideal LS7 metrics in Finnish Public Sector study due to unavailable data on diet.

As shown in Table 3, in WHII, the adjusted HR (95% CI) of multimorbidity for moderate and high level of LE8 score at baseline respectively were 0.58 (0.50–0.66) and 0.41 (0.35–0.48) when compared to low level. The hazard of multimorbidity decreased by 23% (HR 0.77, 95% CI 0.74–0.79) per 10 points higher LE8 score.

**Table 3:** Associations of baseline and 10-year change in LE8 score and of baseline and 4-year change in ideal LS7^+^ metrics with multimorbidity status during follow-up, respectively in the Whitehall II study and Finnish Public Sector study population.

	n MM/N	IR per 1000 PY	HR	95% CI
**Whitehall II study**				
**Baseline**				
Categories of LE8 score points				
Low (0–49)	254/549	18.8	Ref	
Moderate (50–79)	2138/7177	10.5	0.58	0.50–0.66
High (80–100)	368/2003	6.1	0.41	0.35–0.48
Per 10 points LE8 score	2760/9729		0.77	0.74–0.79
**10-year change from baseline to 1997**–**1999**				
Change in categories of LE8 score points				
Persistently low (0–49)	23/43	34.9	Ref	
Low (0–49) → Moderate (50–79) or High (80–100)	23/59	23.3	0.49	0.28–0.88
Moderate (50–79) → Low (0–49)	57/165	20.1	0.62	0.38–1.02
Persistently moderate (50–79)	578/2008	15.7	0.39	0.25–0.59
Moderate (50–79) → High (80–100)	41/185	11.7	0.27	0.16–0.45
High (80–100) → Moderate (50–79) or Low (0–50)	106/533	10.3	0.32	0.21–0.51
Persistently High (80–100)	48/277	8.7	0.27	0.16–0.44
Per 10 points LE8 score increase^a^	876/3270		0.86	0.81–0.93
**Finnish Public Sector study**				
**Baseline**				
No. of ideal LS7^+^ metrics				
0–1	52/202	23.0	Ref	
2	202/950	17.5	0.73	0.54–0.99
3	697/4081	13.2	0.61	0.46–0.81
4	1467/11,373	9.7	0.49	0.37–0.65
5	1879/20,289	6.9	0.38	0.29–0.50
6	1502/23,482	4.8	0.29	0.22–0.38
7	631/14,549	3.2	0.23	0.17–0.30
Per one ideal LS7^+^ metric	6430/74,926		0.78	0.77–0.80
**4-year change from baseline to re-assessment**				
Change in categories of no. of ideal LS7^+^ metrics				
Persistently poor (0–2)	74/430	20.7	Ref	
Poor (0–2) → Intermediate or High (3–7)	49/284	19.8	1.00	0.69–1.43
Intermediate (3–4) → Poor (0–2)	136/832	18.2	0.89	0.67–1.18
Persistently intermediate (3–4)	783/6397	12.8	0.72	0.56–0.91
Intermediate (3–4) → High (5–7)	244/2888	8.6	0.53	0.41–0.69
High (5–7) → Poor (0–2)	27/187	15.8	0.84	0.54–1.31
High (5–7) → Intermediate (3–4)	587/5423	11.4	0.68	0.53–0.86
Persistently high (5–7)	1818/35,333	5.3	0.37	0.29–0.47
Per one ideal LS7^+^ metric increase^b^	3718/51,774		0.80	0.77–0.82

CI, confidence interval; HR, hazard ratio; IR, incidence rate, LE8, Life's Essential 8; MM, multimorbidity; PY, person-years.

The baseline examination took place between 1985 and 1988 in the Whitehall II study and in 2000–2002, 2004–2005, and 2008–2009 in the Finnish Public Sector study (open cohort).

LS7^+^ corresponds to ideal LS7 metrics without healthy diet but with sleep duration.

Hazard ratios and 95% confidence intervals from Cox's proportional hazards models with age as time scale and birth cohort strata, adjusted for sex, education, occupation, and marital status in Finnish Public Sector study and additionally for ethnicity in Whitehall II study.

a Analysis is further adjusted for baseline LE8 score.

b Analysis is further adjusted for baseline ideal LS7^+^ metrics.

The analysis of change in CVH was conducted in 7323 WHII participants and 52,271 FPS participants (flow charts in Supplementary Figures S3 and S4 in the Appendix). There were only minor differences in characteristics between included and eligible participants in the two cohorts (Supplementary Table S8 in the Appendix). Over a median (IQR) 26.5 (22.4–27.0) and 11.6 (7.3–12.2) years of follow-up, 2057 and 3762 multimorbidity events occurred (incidence rate [95% CI] of 11.89 [11.3–12.4] and 7.4 [7.2–7.7] events per 1000 person-years) in WHII and FPS, respectively.

**Fig. 1:**
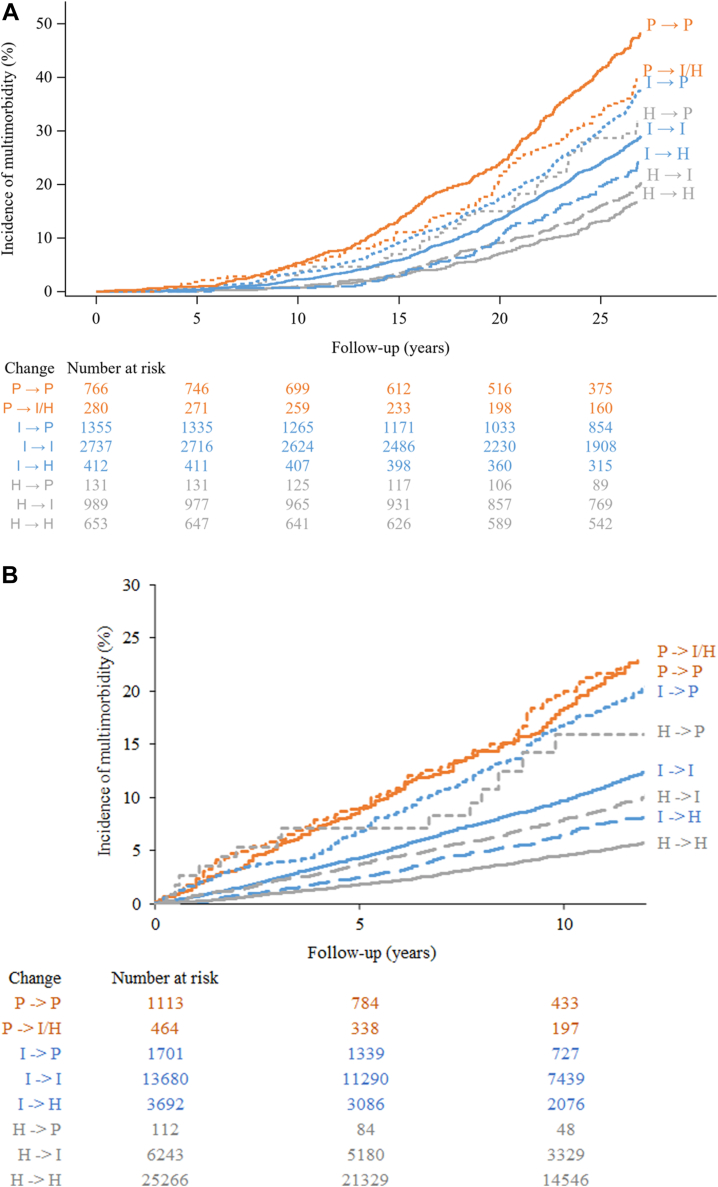
**Cumulative incidence curves for multimorbidity status by category of change in the number of ideal LS7 metrics**. Part A shows incidence by 5-year change from baseline to 1991–1993 in the Whitehall II study population. Part B shows incidence by 4-year change from baseline to second evaluation in the Finnish Public Sector study population. Baseline examination took place in 1985–88 and re-assessment in 1991–1993 in the Whitehall II study with a follow-up of multimorbidity until 31 March 2019. Baseline evaluation took place in 2000–2002, 2004–2005, and 2008–2009 and re-assessment 4 years later in 2004–2005, 2008–2009, and 2012–2013 in the Finnish Public Sector study (open cohort) with a follow-up of multimorbidity until 31 December 2016. LS7 is without healthy diet metric in the Finnish Public Sector study. At baseline and re-assessments, poor (P), intermediate (I), and high (H) LS7 categories defined as 0–2, 3–4, and 5–7 ideal LS7 metrics in the Whitehall II study and as 0–2, 3–4, and 5–6 ideal LS7 metrics in the Finnish Public Sector study. Due to the small number of participants, we combined poor to intermediate or high LS7 category of change. H, high; I, intermediate; P, poor.

**Fig. 2:**
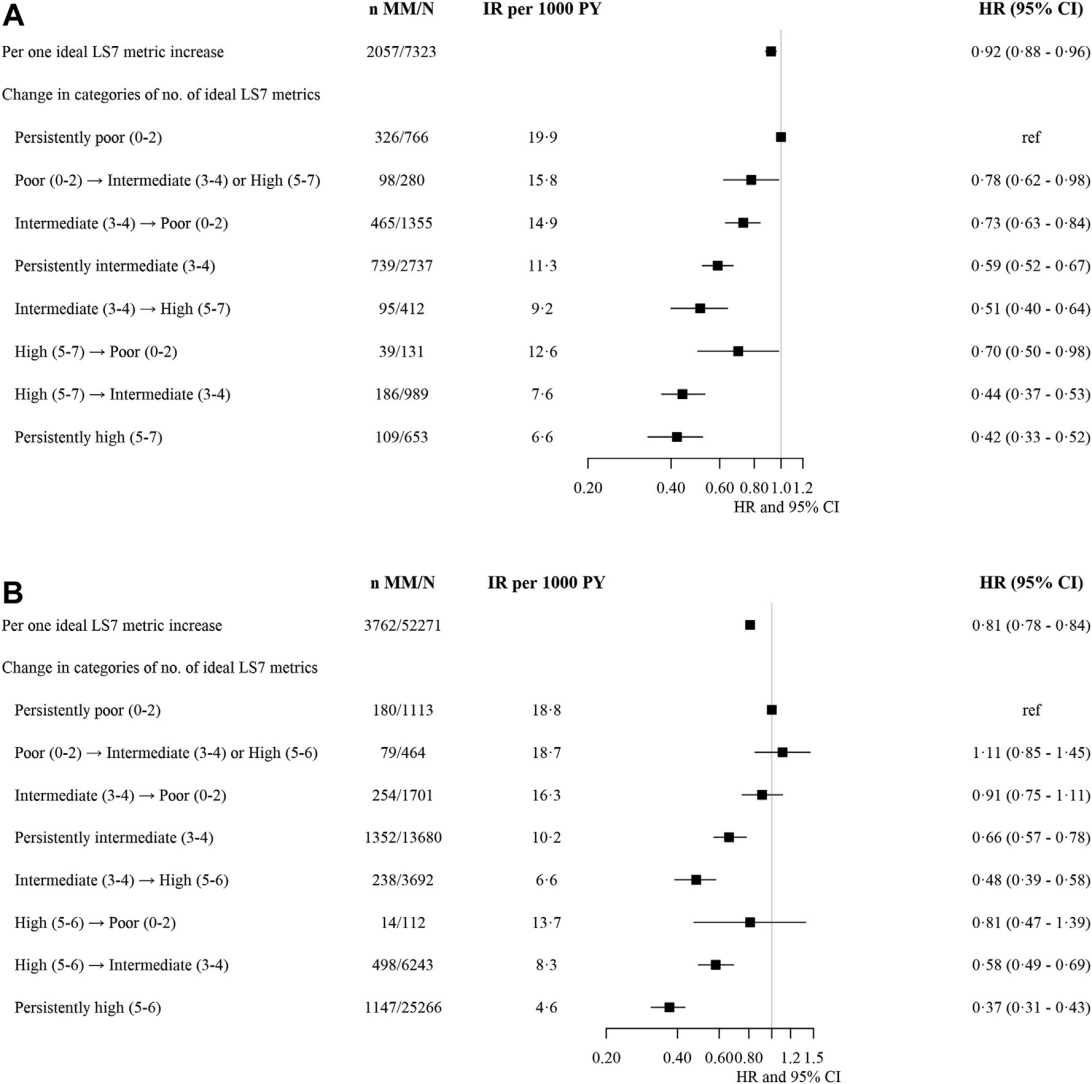
**Hazard ratios and 95% confidence intervals for associations of change in ideal LS7 metrics with multimorbidity status during follow-up**. Part A shows associations of 5-year change from baseline to 1991–1993 in the Whitehall II study population. Part B shows associations of 4-year change from baseline to re-assessment in the Finnish Public Sector study population. Baseline examination took place in 1985–88 and re-assessment in 1991–1993 in the Whitehall II study with a follow-up of multimorbidity until 31 March 2019. Baseline evaluation took place in 2000–2002, 2004–2005, and 2008–2009 and re-assessment 4 years later in 2004–2005, 2008–2009, and 2012–2013 in the Finnish Public Sector study (open cohort) with a follow-up of multimorbidity until 31 December 2016. Hazard ratios and 95% confidence intervals from Cox's proportional hazards models with age as time scale and birth cohort strata, adjusted for sex, education, occupation, and marital status in the Finnish Public Sector study and additionally for ethnicity in the Whitehall II study. Hazard ratios per one ideal LS7 metric increase over time are additionally adjusted for baseline value. LS7 is without healthy diet metric in the Finnish Public Sector Study. At baseline and re-assessments, poor, intermediate, and high LS7 categories defined as 0–2, 3–4, and 5–7 ideal LS7 metrics in the Whitehall II study and as 0–2, 3–4, and 5–6 ideal LS7 metrics in the Finnish Public Sector study. Due to the small number of participants, we combined poor to intermediate or high LS7 category of change. CI, confidence interval; IR, incidence rate; HR, hazard ratio; MM, multimorbidity; PY, person-years.

As shown in Fig. 1, there was a consistent relationship between the direction of the change in categories of CVH and the cumulative incidence of multimorbidity in both WHII and FPS (p < 0.0001 for the difference between curves in both studies). Fig. 2 presents forest plots showing HR and 95% CI for associations of change in ideal LS7 metrics with multimorbidity status during follow-up in the two cohorts. In WHII, change from poor to intermediate or high categories (HR 0.78, 95% CI 0.62–0.98) as well as persistently intermediate (HR 0.59, 95% CI 0.52–0.67) and high (HR 0.42, 95% CI 0.33–0.52) categories were associated with lower multimorbidity hazard when compared to persistently poor category. The hazard of multimorbidity decreased by 8% (HR 0.92, 95% CI 0.88–0.96) per one ideal LS7 metric increase over time. Consistent results were observed in FPS.

As shown in Table 3, the analysis of change in LE8 score in WHII yielded HR (95% CI) of multimorbidity of 0.49 (0.28–0.88) for improvement from low to moderate or high LE8 level, 0.39 (0.25–0.59) for persistently moderate and 0.27 (0.16–0.44) for persistently high LE8 level, when compared to persistently low level. The hazard of multimorbidity decreased by 14% per 10 points LE8 score increase (HR 0.86, 95% CI 0.81–0.93) over time.

In secondary analyses, the PPF (95% CI) for achieving ≥2 ideal LS7 metrics was 53.0% (44.7–59.9) in WHII and 52.9% (42.7–61.2) in FPS. Both in WHII and FPS, the odds of multimorbidity severity decreased per number of ideal LS7 metric at baseline and per increase in the number of ideal LS7 metrics from baseline to re-assessment (Supplementary Table S9 in the Appendix). Furthermore, associations with multimorbidity status remained consistent when examining baseline and change in LS7 score in WHII (Supplementary Table S10 in the Appendix) and baseline and change in ideal LS7^+^ metrics in FPS (Table 3). Except diet, the remaining LS7 metrics and sleep duration were associated with multimorbidity status in the baseline and CVH change analysis (Supplementary Tables S11 and S12 in the Appendix).

We conducted several sensitivity analyses in WHII. Associations of ideal LS7 metrics and LE8 score with incident multimorbidity status were consistent when correcting for cohort attrition (Supplementary Table S13), accounting for competing risks (Supplementary Tables S14 and S15) although effect sizes were attenuated, excluding single chronic diseases at study entry and censoring follow-up after 15 years (Supplementary Tables S16 and S17), excluding cardiovascular disease from multimorbidity (Supplementary Tables S18 and S19 in the Appendix), and when further adjusting for the post-hoc variables (Supplemental Tables S20 and S21).

## Discussion

In two community-based cohort studies, better CVH and improvement in CVH, evaluated by the American Heart Association LS7 and LE8, were associated with lower risk of incident multimorbidity defined as the presence of two or more of 12 chronic diseases. In the primary analysis, the risk of multimorbidity decreased by 8% per one ideal LS7 metric increase over 5 years and by 14% per 10-point increase in LE8 score over 10 years. These results were robust to comprehensive sensitivity analysis and confirmed in terms of change in CVH over 4 years in the validation analysis based on an independent study population.

To our knowledge, this is the first study to investigate associations of change in CVH with multimorbidity risk. In the UK Biobank, better CVH at baseline was found to be associated with longer life expectancy free of four chronic diseases.^17^ We extend this evidence by considering a range of 12 chronic conditions and by evaluating associations of change in CVH with multimorbidity risk. We also extend findings of a recent study that showed a cross-sectional association between higher LE8 score and lower odds of multimorbidity in the National Health and Nutrition Examination Survey from 2007 to 2018.^28^ Our findings on individual LS7 metrics, in particular smoking, physical activity, and body mass index, are in agreement with prior studies on specific factors.^29^, ^30^, ^31^ In one study, healthy diet was associated with risk of transition from cancer but not from cardiovascular disease to multimorbidity.^29^ Coronary heart disease was the most frequent first diagnosis in WHII, which might explain the lacking association of diet with multimorbidity in our study.

There is currently no universally accepted or definitive definition for multimorbidity. Our list was defined using the literature in this domain with the conditions that the list should include at least 12 common conditions to have sufficient coverage^32^ and the conditions should reflect dysfunction in multiple organs.^33^ Too large a list of conditions makes the concept meaningless for clinicians and for identifying prevention targets. For example, a recent paper on the multimorbidity frailty index included 38 conditions, including for example cough, dizziness and giddiness, as well as cerebrovascular disease,^34^ leading to more than 70% of older adults being multimorbid. We chose to concentrate on 13 chronic diseases (12 conditions and diabetes in the descriptive analysis) that cover a large set of body functions and have been previously used in the literature.^33^^,^^35^

The present results have important practical implications, suggesting that improving CVH of the community may contribute to multimorbidity prevention. The lower risk of multimorbidity associated with persistently intermediate/moderate and high as well as improved CVH, together with the lower multimorbidity severity associated with the higher number of ideal LS7 metrics, support the notion that promoting CVH could lower the burden of multimorbidity. Highlighting the potential impact of these strategies, the population preventable fractions in the WHII and FPS studies suggested that half of multimorbidity cases in individuals with fewer than 2 ideal LS7 metrics could have been prevented if they had achieved ≥2 ideal LS7 metrics. Replication of results across two independent cohort studies supports transferability of our findings to communities that differ in terms of context, characteristics, and outcome, including the composition of multimorbidity, as coronary heart disease prevailed in WHII and depression in FPS. Furthermore, approaches targeting CVH may be applicable to both clinical and non-clinical settings as we obtained consistent findings using measured health factors in the primary analysis and self-reports and pharmacy claims in the validation. Notably, self-reported LS7 metrics may be a reliable alternative to measured metrics.^36^

The study has several limitations. Our main analyses were adjusted for age, sex, ethnicity, education, occupation, and marital status. However, residual confounding cannot be excluded, although further adjustment for post-hoc covariates did not impact the results. Given the observational design of our study, the reported association between CVH and multimorbidity should not be considered as being causal. In the FPS study, the level of the biological metrics was defined solely based on records of drugs dispensation and physician diagnosis, as direct clinical measurement data were not available. Therefore, part of individuals classified as achieving an ideal level for a given metric may actually be at an intermediate (or even poor) level. This misclassification may have, if anything, led to an underestimation of the true association between cardiovascular health and the risk of multimorbidity. Data on healthy diet were not available in the FPS study; however, the diet metric was not associated with multimorbidity in the WHII study. Further, in the WHII study, sleep duration was included in the 10-year but not in the 5-year reassessment. For this reason, unlike LS7, it was not possible to measure 5-year change in LE8. However, in the FPS study, the analyses of change in CVH with and without sleep duration were based on the same time interval for re-assessment. These analyses replicated the findings observed in the WHII study, suggesting that the different time frames for measuring LS7 and LE8 in that study did not introduce significant bias to the results. The WHII study is over-represented for males and graduates, does not include manual or similar occupations, and is under-represented for non-White people, limiting generalizability to other social and ethnic groups. However, our analyses were adjusted for sex, education, occupation, and ethnicity. Furthermore, we replicated the main findings in the FPS study which is over-represented for females and includes manual occupations. In a random sample of the Finnish adult population in 2007, the prevalence of 5–7 ideal LS7 metrics was 8.8% in women and 3.0% in men, while the prevalence of 0–2 ideal LS7 metrics was 23.2% in women and 38.5% in men.^37^ Direct comparison of the results in the FPS study with these national data is limited due to the absence of clinical measurement and diet data in FPS. Baseline survey participation was 73% in the WHII study. Of the alive baseline participants, 87% attended to the 5-year re-assessment and 74% to the 10-year re-assessment. Response rates varied between 67% and 69% in the FPS study. Non-response might contribute to overestimation or underestimation of true associations between LS7, LE8 and multimorbidity, although substantial bias is less likely to be introduced by reduced participation than by a large number of dropouts during follow-up, which was largely avoided. Twelve chronic conditions are in the lower range of the distribution of diseases listed for defining multimorbidity in the literature, and less medically-focused conditions were not considered in the current study.

In conclusion, better CVH and its improvement, assessed by the AHA LS7 and LE8, were consistently associated with decreased risk of multimorbidity in two community-based cohort studies. Improving CVH of the community may contribute to multimorbidity prevention.

## Contributors

All authors participated in critically reviewing the analysis and the report and interpreting the findings. J-PE generated the hypothesis and designed the study. M-CP and JP conducted the statistical analysis, and they assessed and verified the data. CP wrote the report. CP and J-PE had full access to WHII data, and MK had full access to FPS data. All authors had access to all the pseudonymised data reported in the study. The corresponding authors had final responsibility for the decision to submit for publication.

## Data sharing statement

The data underlying this study can be assessed for research purposes with a signed data access agreement. In the WHII study, the data are available from Dementias Platform UK: https://portal.dementiasplatform.uk/. In the FPS study, questionnaire data are available by request to the investigators (jenni.ervasti@ttl.fi). Access to linked register data requires separate permissions from the register keepers (Finnish Institute of Health and Welfare, Social Insurance Institution of Finland and Statistics Finland).

## Declaration of interests

The authors declare no competing interests.
