# Supplementary material for: Association between changes in cardiovascular health and the risk of multimorbidity: community-based cohort studies in the UK and Finland
Source: Lancet Reg Health Eur. 2024 May 6;42:100922. doi: 10.1016/j.lanepe.2024.100922 (PMC11098950; doi:10.1016/j.lanepe.2024.100922)
Supplement: Supplementary Tables and Figures [file mmc1.docx]

**SUPPLEMENTARY APPENDIX**

Supplement to: Prugger C, Perier MC, Sabia S, Fayosse A, van Sloten TT, Jouven X, Pentti J, Kivimäki M, Empana JP. Association between changes in cardiovascular health and the risk of multimorbidity: community-based cohort studies in the UK and Finland.

**Table of contents**

[Filled in STROBE checklist 3](#_Toc160052382)

[Supplementary Table 1 4](#_Toc160052383)

[Supplementary Table 2 5](#_Toc160052384)

[Supplementary Table 3 7](#_Toc160052385)

[Supplementary Table 4 8](#_Toc160052386)

[Supplementary Table 5 9](#_Toc160052387)

[Supplementary Table 6 10](#_Toc160052388)

[Supplementary Table 7 11](#_Toc160052389)

[Supplementary Table 8 12](#_Toc160052390)

[Supplementary Table 9 13](#_Toc160052391)

[Supplementary Table 10 14](#_Toc160052392)

[Supplementary Table 11 15](#_Toc160052393)

[Supplementary Table 12 16](#_Toc160052394)

[Supplementary Table 13 17](#_Toc160052395)

[Supplementary Table 14 18](#_Toc160052396)

[Supplementary Table 15 19](#_Toc160052397)

[Supplementary Table 16 20](#_Toc160052398)

[Supplementary Table 17 21](#_Toc160052399)

[Supplementary Table 18 22](#_Toc160052400)

[Supplementary Table 19 23](#_Toc160052401)

[Supplementary Table 20 24](#_Toc160052402)

[Supplementary Table 21 25](#_Toc160052403)

[Supplementary Figure 1 26](#_Toc160052404)

[Supplementary Figure 2 27](#_Toc160052405)

[Supplementary Figure 3 28](#_Toc160052406)

[Supplementary Figure 4 30](#_Toc160052407)

[Data access application 31](#_Toc160052408)

[Analysis plan for replication 32](#_Toc160052409)

[Study protocol 33](#_Toc160052410)

[Statistical code 34](#_Toc160052411)

[References 51](#_Toc160052412)

# **Filled in STROBE checklist**

|  | Item No | Recommendation | Page  No |
| --- | --- | --- | --- |
| **Title and abstract** | 1 | (*a*) Indicate the study’s design with a commonly used term in the title or the abstract | 1 |
|  |  | (*b*) Provide in the abstract an informative and balanced summary of what was done and what was found | 3 |
| Introduction | | |  |
| Background/rationale | 2 | Explain the scientific background and rationale for the investigation being reported | 7 |
| Objectives | 3 | State specific objectives, including any prespecified hypotheses | 7 |
| Methods | | |  |
| Study design | 4 | Present key elements of study design early in the paper | 8 |
| Setting | 5 | Describe the setting, locations, and relevant dates, including periods of recruitment, exposure, follow-up, and data collection | 8, 9 |
| Participants | 6 | (*a*) Give the eligibility criteria, and the sources and methods of selection of participants. Describe methods of follow-up | 8, 9 |
|  |  | (*b*) For matched studies, give matching criteria and number of exposed and unexposed |  |
| Variables | 7 | Clearly define all outcomes, exposures, predictors, potential confounders, and effect modifiers. Give diagnostic criteria, if applicable | 9, 10 |
| Data sources/ measurement | 8* | For each variable of interest, give sources of data and details of methods of assessment (measurement). Describe comparability of assessment methods if there is more than one group | 8, 9, 10 |
| Bias | 9 | Describe any efforts to address potential sources of bias | 11 |
| Study size | 10 | Explain how the study size was arrived at | 12 |
| Quantitative variables | 11 | Explain how quantitative variables were handled in the analyses. If applicable, describe which groupings were chosen and why | 10, 11 |
| Statistical methods | 12 | (*a*) Describe all statistical methods, including those used to control for confounding | 10, 11 |
|  |  | (*b*) Describe any methods used to examine subgroups and interactions | NA |
|  |  | (*c*) Explain how missing data were addressed | 11 |
|  |  | (*d*) If applicable, explain how loss to follow-up was addressed | 11 |
|  |  | (*e*) Describe any sensitivity analyses | 11 |
| Results | | |  |
| Participants | 13* | (a) Report numbers of individuals at each stage of study—eg numbers potentially eligible, examined for eligibility, confirmed eligible, included in the study, completing follow-up, and analysed | Suppl. 28-30 |
|  |  | (b) Give reasons for non-participation at each stage | Suppl. 28-30 |
|  |  | (c) Consider use of a flow diagram | Suppl. 28-30 |
| Descriptive data | 14* | (a) Give characteristics of study participants (eg demographic, clinical, social) and information on exposures and potential confounders | 12, 19 |
|  |  | (b) Indicate number of participants with missing data for each variable of interest | 19 |
|  |  | (c) Summarise follow-up time (eg, average and total amount) | 13 |
| Outcome data | 15* | Report numbers of outcome events or summary measures over time | 13, 20, 21 |
| Main results | 16 | (*a*) Give unadjusted estimates and, if applicable, confounder-adjusted estimates and their precision (eg, 95% confidence interval). Make clear which confounders were adjusted for and why they were included | 13, 20, 21 |
|  |  | (*b*) Report category boundaries when continuous variables were categorized | 19, 20, 21 |
|  |  | (*c*) If relevant, consider translating estimates of relative risk into absolute risk for a meaningful time period | 13, 20, 21 |
| Other analyses | 17 | Report other analyses done—eg analyses of subgroups and interactions, and sensitivity analyses | 13, 14 |
| Discussion | | |  |
| Key results | 18 | Summarise key results with reference to study objectives | 14 |
| Limitations | 19 | Discuss limitations of the study, taking into account sources of potential bias or imprecision. Discuss both direction and magnitude of any potential bias | 15 |
| Interpretation | 20 | Give a cautious overall interpretation of results considering objectives, limitations, multiplicity of analyses, results from similar studies, and other relevant evidence | 15 |
| Generalisability | 21 | Discuss the generalisability (external validity) of the study results | 15 |
| Other information | | |  |
| Funding | 22 | Give the source of funding and the role of the funders for the present study and, if applicable, for the original study on which the present article is based | NA |

Page numbers in the manuscript submitted, unless otherwise stated.

*Give information separately for exposed and unexposed groups.

Supplementary Table 1

**Definitions of the LS7 metrics for the assessment of cardiovascular health at baseline (1985-1988) and in 1991-1993 in the Whitehall II study population**

| **Metric** | **Recommended ideal level** | **Intermediate level** | **Poor level** |
| --- | --- | --- | --- |
| Smoking | Never or quit ≥12 months | Quit <12 months | Current smoker |
| Physical activity^a^ | ≥75 min/week of vigorous activity, ≥150 min/week of moderate activity or a combination of the two | 1–74 min/week of vigorous activity, 1–149 min/week of moderate activity or a combination of the two | None |
| Healthy diet^b^ | At baseline:  ≥2 optimal out of the following 3 items: 2 servings of a fruit and vegetable portion/day, fish consumption ≥3 times/week, and consumption of fiber-rich aliments content ≥3 times/week.  In 1991-1993:  ≥4 optimal out of the following 5 items: ≥2 servings of a fruit and vegetable portion/day, fish consumption ≥3 times/week, consumption of fiber-rich aliments content ≥3 times/day, sodium consumption ≤1500 mg/day, and ≤450 kcal of sugar-sweetened beverages/week | At baseline:  1 optimal out of the 3 items  In 1991-1993:  2-3 optimal out of the 5 items | At baseline:  0 optimal out of the 3 items  In 1991-1993:  0-1 optimal out of the 5 items |
| Body mass index | BMI <25 kg/m^2^ | 25-29.9 kg/m^2^ | ≥30 kg/m^2^ |
| Blood pressure^c^ | <120/80 mmHg, untreated | <120/80 mmHg on medications or  120-139/80-89 mmHg | ≥140/90 mmHg |
| Fasting blood glucose^d^ | 100 mg/dL, untreated | 100-126 mg/dL or  <100 mg/dL treated | >126 mg/dL |
| Total cholesterol^d^ | <200 mg/dL, untreated | 200-240 mg/dL or  <200 mg/dL treated | >240 mg/dL |

Each LS7 metric is weighted by 0, 1, or 2 to reflect poor, intermediate, and ideal level, respectively, permitting to consider the number of ideal metrics (range 0 to 7), the LS7 score (range 0 to 14 points), and poor, intermediate, and high CVH defined by 0-2, 3-4, and 5-7 ideal metrics or 0-5, 6-9, and 10-14 points.

^a^Physical activity was assessed using questions on frequency and duration of participation in mildly energetic (e.g., weeding, general housework, bicycle repair), moderately energetic (e.g., dancing, cycling, leisurely swimming), and vigorous physical activity (e.g., running, hard swimming, playing squash).

^b^For the diet metric at baseline (1985-1988), only the frequency of fruits, vegetables, and fish consumption was reported. In 1991-1993, the frequency consumption of the 5 AHA-recommended items was drawn from a validated 127-item food frequency questionnaire.

^c^Systolic blood pressure was measured twice with a sphygmomanometer in the sitting position after 5 min rest, and the average of the two readings was used in the present analyses. Participants reported their medications used in the previous 14 days; responses were coded using the British National Formulary.

^d^Total cholesterol and fasting blood glucose were measured using standardized methods. Participants reported their medications used in the previous 14 days; responses were coded using the British National Formulary. At baseline (1985-1988), however, only prevalent diabetes and antidiabetic medications were available.

SI conversion factor: To convert glucose to millimoles per liter, multiply by 0.0555.

SI conversion factor: To convert cholesterol to millimoles per liter, multiply by 0.0259.

Supplementary Table 2

**Definitions of the LE8 metrics for the assessment of cardiovascular health at baseline (1985-1988) and in 1997-1999 in the Whitehall II study population**

| **Metric** | **Assessment** | **Scoring** |
| --- | --- | --- |
| Nicotine exposure | Based on smoking status and age when stopped smoking. No data on secondhand smoke exposure nor nicotine- delivery system. | Points Status  100 Never smoker  75 Former smoker, quit ≥5 y  50 Former smoker, quit 1–<5 y  25 Former smoker, quit <1 y  0 Current smoker |
| Physical activity | Based on minutes of moderate and vigorous activity (with vigorous equal 2 minutes of moderate activity). | Points Minutes  100 ≥150  90 120–149  80 90–119  60 60–89  40 30–59  20 1–29  0 0 |
| Healthy diet | Baseline:  Based on fruits and vegetables, whole grain (fibers in bread, and cereals), and red and processed meat.  1997-1999:  Based on fruits, vegetables, nuts and legumes, whole grain (fibers in nutrient analysis of FFQ food intake), low-fat dairy, sodium, red and processed meat, and sweetened beverages. | Points Quantile  100 ≥95th percentile (top/ideal  diet)  80 75th–94th percentile  50 50th–74th percentile  25 25th–49th percentile  0 1st–24th percentile (bottom/  least ideal quartile) |
| Sleep duration | Based on self-reported sleep duration with adjustment because of categorized data. | Points Hours  100 8  90 >9  70 7  40 6  10 <=5 |
| Blood pressure | Based on measured systolic/ diastolic blood pressure and blood pressure lowering treatment. | Points Level (mmHg)  100 <120/<80  75 120–129/<80  50 130–139 or 80–89  25 140–159 or 90–99  0 ≥160 or ≥100  Subtracted 20 points if treated level |
| Body mass index | Based on body weight in kilograms divided by height in meters squared. | Points Level (kg/m²)  100 <25  70 25.0–29.9  30 30.0–34.9  15 35.0–39.9  0 ≥40.0 |
| Fasting blood glucose | Baseline:  Based on diabetes and antidiabetic medication.  1997-1999:  Based on fasting blood glucose, with conversion for the 5 first levels as described in Glucose Management Indicator (GMI): A New Term for Estimating A1C From Continuous Glucose Monitoring. ^1^ | Points Level (mg/dL)  Baseline:  100 No diabetes and no treatment  40 Treatment  10 Diabetes and no treatment  1997-1999:  100 No history of diabetes and FBG<100  60 No history of diabetes and 100≤FBG<126  40 Diabetes with FBG<154  30 Diabetes with 154≤FBG<196  20 Diabetes with 196≤FBG<238  10 Diabetes with 238≤FBG<280  0 Diabetes with 280≤FBG |
| Lipids | Baseline:  Based on total cholesterol level and data on lipid-lowering medication.  1997-1999:  Based on non-HDL cholesterol level and data on lipid-lowering medication. | Points Level (mg/dL)  Baseline:  80 <200  50 200-<240  10 ≥240  Subtracted 20 points, if drug-treated level  1997-1999:  100 <130  60 130–159  40 160–189  20 190–219  0 ≥220  Subtracted 20 points, if drug-treated level |

Each LE8 metric is scored from 0 to 100 and their sum is averaged to derive the LE8 score (range 0 to 100 points), from which low (0-49), moderate (50-79), and high (80-100) CVH can be defined.

SI conversion factor: To convert glucose to millimoles per liter, multiply by 0.0555.

SI conversion factor: To convert cholesterol to millimoles per liter, multiply by 0.0259.

FBG, fasting blood glucose

Supplementary Table 3

**Definitions of the LS7 metrics for the assessment of cardiovascular health at baseline and second evaluation in the Finnish Public Sector study population**

| **Metric** | **Ideal level** | **Non-ideal level** |
| --- | --- | --- |
| Smoking | Never smoking | Current or ever smoking |
| Physical activity^a^ | ≥14 MET-h/week | <14 MET-h/week (according to recommendations by WHO) ^2^ |
| Healthy diet | NA | NA |
| Body mass index^b^ | <25 kg/m^2^ | ≥25 kg/m^2^ |
| Blood pressure^c^ | No hypertension diagnosis and untreated | Hypertension diagnosis and/or treated |
| Fasting blood glucose^c^ | No diabetes diagnosis and untreated | Diabetes diagnosis and/or treated |
| Total cholesterol^d^ | Untreated | Treated |

Each LS7 metric is weighted as 0 or 1 to reflect non ideal vs. ideal level. In the absence of the diet metric, the number of ideal metrics can range from 0 to 6, with poor, intermediate, and high CVH defined by 0-2, 3-4, and 5-6 ideal metrics.

^a^Physical activity was assessed using questions on average weekly hours of both leisure time and commuting physical activity using four different intensity levels: walking, brisk walking, jogging and running (or other activities with a corresponding intensity level).

^b^Body mass index was calculated using self-reported height and weight.

^c^Blood pressure and fasting blood glucose metric were assessed using data on hypertension and diabetes diagnosis from self-reports and data on blood pressure lowering and antidiabetic treatment from pharmacy claims.

^d^Total cholesterol metric was assessed using data on lipid lowering treatment from pharmacy claims.

NA, not available; WHO, World Health Organization

Supplementary Table 4

**Data and criteria for ascertaining the 12 chronic diseases of multimorbidity in the Whitehall II study and Finnish Public Sector study populations**

|  | **Whitehall II study** | **Finnish Public Sector study** | | |
| --- | --- | --- | --- | --- |
| **Disease** | **Hospital Episodes Statistics, ICD-10** | **Hospital Discharge Register (in-patient),  Death Register, ICD-10** | **Drug Reimbursement Register** | **Purchase  Register, ATC** |
| Cancer | C00-C97 | C00-C97 |  |  |
| CHD | I20-I25 (and 12-lead resting ECG recording) | I20-I25 | X |  |
| Stroke | I60-I64 (and MONICA-Augsburg stroke questionnaire) | I60-I64 |  |  |
| Heart failure | I50 | I50 | X |  |
| COPD | J41-J44 | J41-J44 | X |  |
| Chronic kidney disease | N18 | N18 | X |  |
| Liver disease | K70-K74 | K70-K74 |  |  |
| Depression | F32, F33, or use of antidepressants | F32,F33 |  | N06A |
| Dementia | F00-F03, F05.1, G30, G31 | F00-F03,F05.1,G30,G31 | X |  |
| Mental disorders, other than depression and dementia | F06, F07, F09, F20-F31, F34-48, F60-F64, F67-F69 | F06, F07, F09, F20-F31, F34-F48, F60-F64, F67-F69 |  |  |
| Parkinson’s disease | G20 | G20 | X |  |
| Arthritis/rheumatoid arthritis | M15-M19, M05, M06 | M15-M19,M05,M06 | X |  |

CHD, coronary heart disease; COPD, chronic obstructive pulmonary disease; ECG, electrocardiogram; ICD, International Classification of Diseases

Supplementary Table 5

**Baseline characteristics of the Whitehall II study participants by categories of LE8 score points and of the Finnish Public Sector study participants by categories of** **number of ideal LS7+ metrics** **additionaly including sleep duration**

| **Whitehall II study (N=9729)** | | | | **Finnish Public Sector study (N=74926)** | | | |
| --- | --- | --- | --- | --- | --- | --- | --- |
|  | **Low (0-49)** | **Moderate (50-79)** | **High (80-100)** |  | **Poor (0-2)** | **Intermediate (3-4)** | **High (5-7)** |
|  | **N=549** | **N=7177** | **N=2003** |  | **N=1152** | **N=15454** | **N=58320** |
| Age, mean (SD), y | 47·4 (5·8) | 45.2 (6·0) | 42·8 (5·6) | Age, mean (SD), y | 51·9 (7·9) | 46·9 (9·5) | 42·2 (10·0) |
| Men, n (%) | 293 (53·4) | 4908 (68·4) | 1364 (68·1) | Men, n (%) | 397 (34·5) | 4144 (26·8) | 10562 (18·1) |
| Education level, n (%) |  |  |  | Education level, n (%) |  |  |  |
| Lower secondary school or less | 224 (40·8) | 1918 (26·7) | 356 (17·8) | Primary | 242 (21·0) | 2342 (15·2) | 4173 (7·2) |
| Higher secondary school | 84 (15·3) | 1353 (18·9) | 363 (18·1) | Secondary | 458 (39·8) | 6148 (39·8) | 18313 (31·4) |
| University or higher degree | 96 (17·5) | 2141 (29·8) | 772 (38·5) | Tertiary or higher | 452 (39·2) | 6964 (45·1) | 35834 (61·4) |
| Unknown | 145 (26·4) | 1765 (24·6) | 512 (25·6) |  |  |  |  |
| Occupation, n (%) |  |  |  | Occupation, n (%) |  |  |  |
| Administrative | 64 (11·7) | 2096 (29·2) | 740 (36·9) | Low | 557 (48·4) | 6932 (44·9) | 19116 (32·8) |
| Professional/ executive | 205 (37·3) | 3506 (48·9) | 1010 (50·4) | Intermediate | 353 (30·6) | 4880 (31·6) | 19877 (34·1) |
| Clerical/support | 280 (51·0) | 1575 (21·9) | 253 (12·6) | High | 242 (21·0) | 3642 (23·6) | 19327 (33·1) |
| Marital status, n (%) |  |  |  | Marital status, n (%) |  |  |  |
| Married/ cohabiting | 342 (62·3) | 5323 (74·2) | 1554 (77·6) | Spouse | 840 (72·9) | 11516 (74·5) | 44350 (76·1) |
| Single | 114 (20·8) | 1172 (16·3) | 309 (15·4) | No spouse | 312 (27·1) | 3938 (25·5) | 13970 (24·0) |
| Divorced | 77 (14·0) | 585 (8·2) | 128 (6·4) |  |  |  |  |
| Widowed | 16 (2·9) | 97 (1·4) | 12 (0·6) |  |  |  |  |
| Ethnicity, white, n (%) | 459 (83·6) | 6460 (90·0) | 1859 (92·8) | Ethnicity | NA | NA | NA |

LE8, Life’s Essential 8; LS7, Life’s Simple 7; NA, not available; SD, standard deviation

Supplementary Table 6

**Six most common combinations of two conditions during follow-up in the Whitehall II study and Finnish Public Sector study populations**

|  | **Whitehall II study** | | | **Finnish Public Sector study** | | |
| --- | --- | --- | --- | --- | --- | --- |
| **Multimorbidity considering 12 conditions** | | | | | | |
| **Baseline analysis** | **N=1464 MM events of two conditions** | | | **N=5630 MM events of two conditions** | | |
| **Rank** | **n** | **First event** | **Second event** | **n** | **First event** | **Second event** |
| 1. | 110 | CHD | Cancer | 1243 | Depression | Arthritis |
| 2. | 95 | CHD | Arthritis | 994 | Depression | Cancer |
| 3. | 89 | Cancer | Arthritis | 614 | Cancer | Depression |
| 4. | 62 | Arthritis | Cancer | 532 | Arthritis | Depression |
| 5. | 58 | Cancer | CHD | 320 | Depression | CHD |
| 6. | 56 | CHD | Heart failure | 313 | Depression | Mental disorder |
| **Change analysis** | **N=1141 MM events of two conditions** | | | **N=3357 MM events of two conditions** | | |
| **Rank** | **n** | **First event** | **Second event** | **n** | **First event** | **Second event** |
| 1. | 87 | CHD | Cancer | 746 | Depression | Arthritis |
| 2. | 81 | CHD | Arthritis | 595 | Depression | Cancer |
| 3. | 68 | Cancer | Arthritis | 366 | Cancer | Depression |
| 4. | 46 | Arthritis | Cancer | 330 | Arthritis | Depression |
| 5. | 44 | Cancer | CHD | 191 | Depression | CHD |
| 6. | 43 | CHD | Heart failure | 136 | Depression | Mental disorder |
| **Multimorbidity considering 12 conditions plus diabetes** | | | | | | |
| **Baseline analysis** | **N=1611 MM events of two conditions** | | | **N=7259 MM events of two conditions** | | |
| **Rank** | **n** | **First event** | **Second event** | **n** | **First event** | **Second event** |
| 1. | 91 | CHD | Diabetes | 1128 | Depression | Diabetes |
| 2. | 89 | CHD | Cancer | 1040 | Depression | Arthritis |
| 3. | 77 | Cancer | Arthritis | 858 | Depression | Cancer |
| 4. | 72 | CHD | Arthritis | 552 | Cancer | Depression |
| 5. | 66 | Diabetes | Cancer | 461 | Diabetes | Depression |
| 6. | 66 | Diabetes | CHD | 447 | Arthritis | Depression |
| **Change analysis** | **N=1269 MM events of two conditions** | | | **N=4331 MM events of two conditions** | | |
| **Rank** | **n** | **First event** | **Second event** | **n** | **First event** | **Second event** |
| 1. | 73 | CHD | Cancer | 650 | Depression | Diabetes |
| 2. | 73 | CHD | Diabetes | 626 | Depression | Arthritis |
| 3. | 64 | CHD | Arthritis | 513 | Depression | Cancer |
| 4. | 59 | Cancer | Arthritis | 329 | Cancer | Depression |
| 5. | 57 | Diabetes | Cancer | 280 | Arthritis | Depression |
| 6. | 49 | Diabetes | CHD | 243 | Diabetes | Depression |

CHD, coronary heart disease; MM, multimorbidity

Supplementary Table 7

**Six most common combinations of three conditions during follow-up in the Whitehall II study and Finnish Public Sector study populations**

|  | **Whitehall II study** | | | | **Finnish Public Sector study** | | | |
| --- | --- | --- | --- | --- | --- | --- | --- | --- |
| **Multimorbidity considering 12 conditions** | | | | | | | | |
| **Baseline analysis** | **N=738 MM events of three conditions** | | | | **N=755 events of three conditions** | | | |
| **Rank** | **n** | **First event** | **Second event** | **Third event** | **N** | **First event** | **Second event** | **Third event** |
| 1. | 19 | CHD | Heart failure | CKD | 68 | Depression | Arthritis | Cancer |
| 2. | 11 | Arthritis | CHD | Heart failure | 39 | Arthritis | Depression | Cancer |
| 3. | 10 | CHD | Cancer | Arthritis | 31 | Arthritis | Cancer | Depression |
| 4. | 10 | CHD | Arthritis | Cancer | 30 | Cancer | Depression | Arthritis |
| 5. | 10 | CHD | Heart failure | COPD | 26 | Depression | Cancer | Arthritis |
| 6. | 8 | Cancer | Arthritis | CHD | 25 | Depression | Arthritis | CHD |
| **Change analysis** | **N=531 MM events of three conditions** | | | | **N=373 events of three conditions** | | | |
| **Rank** | **n** | **First event** | **Second event** | **Third event** | **N** | **First event** | **Second event** | **Third event** |
| 1. | 14 | CHD | Heart failure | CKD | 35 | Depression | Arthritis | Cancer |
| 2. | 10 | CHD | Cancer | Arthritis | 22 | Arthritis | Depression | Cancer |
| 3. | 9 | Arthritis | CHD | Heart failure | 16 | Arthritis | Cancer | Depression |
| 4. | 8 | CHD | Cancer | Heart failure | 15 | Cancer | Depression | Arthritis |
| 5. | 8 | CHD | Arthritis | Cancer | 12 | Depression | Cancer | Arthritis |
| 6. | 8 | CHD | Heart failure | COPD | 12 | Depression | Arthritis | CHD |
| **Multimorbidity considering 12 conditions plus diabetes** | | | | | | | | |
| **Baseline analysis** | **N=841 MM events of three conditions** | | | | **N=1412MM events of three conditions** | | | |
| **Rank** | **n** | **First event** | **Second event** | **Third event** | **n** | **First event** | **Second event** | **Third event** |
| 1. | 15 | CHD | Diabetes | Arthritis | 81 | Depression | Arthritis | Diabetes |
| 2. | 12 | CHD | Heart failure | CKD | 72 | Depression | Diabetes | Arthritis |
| 3. | 11 | CHD | Diabetes | Cancer | 56 | Depression | Diabetes | Cancer |
| 4. | 9 | CHD | Cancer | Arthritis | 52 | Depression | Arthritis | Cancer |
| 5. | 9 | CHD | Diabetes | Heart failure | 47 | Depression | Cancer | Diabetes |
| 6. | 8 | Cancer | CHD | Heart failure | 35 | Arthritis | Depression | Cancer |
| **Change analysis** | **N=608 MM events of three conditions** | | | | **N=697 MM events of three conditions** | | | |
| **Rank** | **n** | **First event** | **Second event** | **Third event** | **n** | **First event** | **Second event** | **Third event** |
| 1. | 12 | CHD | Diabetes | Arthritis | 41 | Depression | Arthritis | Diabetes |
| 2. | 9 | CHD | Cancer | Arthritis | 38 | Depression | Diabetes | Arthritis |
| 3. | 8 | CHD | Heart failure | CKD | 35 | Depression | Cancer | Diabetes |
| 4. | 8 | Depression | CHD | Diabetes | 27 | Depression | Arthritis | Cancer |
| 5. | 7 | Cancer | CHD | Heart failure | 24 | Depression | Diabetes | Cancer |
| 6. | 7 | CHD | Cancer | Heart failure | 19 | Arthritis | Depression | Cancer |

CHD, coronary heart disease; COPD, chronic obstructive pulmonary disease; CKD, chronic kidney disease; MM, multimorbidity

Supplementary Table 8

**Baseline characteristics of included and eligible participants in 1991-1993 in the Whitehall II study population and at re-assessment in the Finnish Public Sector study population**

| **Whitehall II study** | | | **Finnish Public Sector study** | | |
| --- | --- | --- | --- | --- | --- |
|  | **Included (N=7323)** | **Eligible (N=9601)** |  | **Included (N=52271)** | **Eligible (N=83581)** |
| Age, mean (SD), y | 44·7 (6·0) | 44·8 (6·0) | Age, mean (SD), y | 43·8 (9·8) | 43·2 (10·1) |
| Men, n (%) | 5114 (69·8) | 6490 (67·6) | Women, n (%) | 42624 (81·5) | 66801 (79·9) |
| Education level, n (%) |  |  | Education level, n (%) |  |  |
| Lower SS or less | 1773 (24·2) | 2470 (25·7) | Primary | 4313 (8·3) | 6630 (8·9) |
| Higher SS | 1381 (18·9) | 1781 (18·6) | Secondary | 16823 (32·2) | 24729 (33·3) |
| University or higher | 2338 (31·9) | 2987 (31·1) | Tertiary or higher | 31135 (59·6) | 42938 (57·8) |
| Unknown | 1831 (25·0) | 2363 (24·6) |  |  |  |
| Occupation, n (%) |  |  | Occupation, n (%) |  |  |
| Administrative | 2331 (31·8) | 2879 (30·0) | Low | 17609 (33·7) | 26366 (35·5) |
| Professional/executive | 3661 (45·0) | 4661 (48·5) | Intermediate | 18110 (34·7) | 24922 (33·5) |
| Clerical/support | 1331 (18·2) | 2061 (21·5) | High | 16552 (31·7) | 23009 (31·0) |
| Marital status, n (%) |  |  | Marital status, n (%) |  |  |
| Married/cohabiting | 5538 (75·6) | 7136 (74·3) | Spouse | 39986 (76·5) | 56285 (75·8) |
| Single | 1149 (15·7) | 1559 (16·2) | No spouse | 12285 (23·5) | 18012 (24·2) |
| Divorced | 555 (7·6) | 785 (8·2) |  |  |  |
| Widowed | 81 (1·1) | 121 (1·3) |  |  |  |
| Ethnicity, white, n (%) | 6676 (91·2) | 8679 (90·4) | Ethnicity | NA | NA |

Eligible participants consist of included participants and participants who dropped out or had missing data; participants with incident multimorbidity and participants who died between baseline and re-assessment are not considered.

MM, multimorbidity; NA, not available; SD, standard deviation; SS, secondary school

Supplementary Table 9

**Associations of ideal LS7 metrics at baseline and its change from baseline to re-assessment with multimorbidity severity during follow-up in the Whitehall II study and Finnish Public Sector study populations**

|  | **Whitehall II study** | | | **Finnish Public Sector study** | | |
| --- | --- | --- | --- | --- | --- | --- |
|  | **N** | **OR** | **95% CI** | **N** | **OR** | **95% CI** |
| **Baseline** |  |  |  |  |  |  |
| Per one ideal LS7 metric |  |  |  |  |  |  |
| 0-1 condition | 6964 | Ref |  | 68902 | Ref |  |
| 2 conditions | 1464 | 0·84 | 0·79-0·88 | 5630 | 0·79 | 0·77-0·81 |
| 3 conditions | 738 | 0·75 | 0·70-0·81 | 755 | 0·68 | 0·64-0·72 |
| 4 or more conditions | 549 | 0·70 | 0·64-0·76 | 90 | 0·58 | 0·49-0·68 |
| **Change from baseline to re-assessment** |  |  |  |  |  |  |
| Per one ideal LS7 metric increase |  |  |  |  |  |  |
| 0-1 condition | 5266 | Ref |  | 48509 | Ref |  |
| 2 conditions | 1141 | 0·93 | 0·87-0·99 | 3356 | 0·84 | 0·80-0·87 |
| 3 conditions | 531 | 0·89 | 0·81-0·97 | 373 | 0·75 | 0·67-0·84 |
| 4 or more conditions | 385 | 0·88 | 0·79-0·98 | 33 | 0·50 | 0·35-0·72 |

Baseline examination took place in 1985-88 and re-assessment in 1991-1993 in the Whitehall II study with a follow-up of multimorbidity until 31 March 2019. Baseline evaluation took place in 2000-2002, 2004-2005, and 2008-2009 and re-assessment 4 years later in 2004-2005, 2008-2009, and 2012-2013 in the Finnish Public Sector study (open cohort) with a follow-up of multimorbidity until 31 December 2016.

Odds ratios and 95% confidence intervals from multinomial logistic regression analysis, adjusted for age, birth cohort, sex, education, occupation, and marital status in Finnish Public Sector study and additionally for ethnicity in Whitehall II study.

CI, confidence interval; LS7, Life’s Simple 7; OR, Odds ratio

Supplementary Table 10

**Associations of LS7 score at baseline and its 5-year change to 1991-1993 with multimorbidity status during follow-up in the Whitehall II study population**

|  | **n MM/N** | **IR per 1000 PY** | **HR** | **95% CI** |
| --- | --- | --- | --- | --- |
| **Baseline** |  |  |  |  |
| LS7 score categories |  |  |  |  |
| 0-5 | 269/528 | 21·0 | Ref |  |
| 6-9 | 1775/5514 | 11·5 | 0·54 | 0·47-0·62 |
| 10-14 | 707/3673 | 6·5 | 0·35 | 0·30-0·41 |
| Per one point LS7 score | 2751/9715 |  | 0·86 | 0·84-0·87 |
| **5-year change from baseline to 1991-1993** | |  |  |  |
| Change in categories of LS7 score |  |  |  |  |
| Persistently poor (0-5) | 89/156 | 29·0 | Ref |  |
| Poor (0-5) → Intermediate or High (6-14) | 72/153 | 23·0 | 0·71 | 0·52-0·97 |
| Intermediate (6-9) → Poor (0-5) | 147/342 | 12·0 | 0·63 | 0·48-0·82 |
| Persistently intermediate (6-9) | 984/2972 | 14·3 | 0·47 | 0·38-0·59 |
| Intermediate (6-9) → High (10-14) | 185/736 | 10·4 | 0·36 | 0·28-0·47 |
| High (10-14) → Intermediate or Poor (0-9) | 248/1145 | 8·9 | 0·32 | 0·25-0·41 |
| Persistently high (10-14) | 332/1819 | 7·3 | 0·29 | 0·23-0·38 |
| Per one point LS7 score increase* | 2057/7323 |  | 0·92 | 0·90-0·95 |

Hazard ratios and 95% confidence intervals from Cox’s proportional hazards models with age as time scale and birth cohort strata, adjusted for sex, education, occupation, and marital status.

*Analysis is further adjusted for baseline LS7 score.

CI, confidence interval; HR, hazard ratio; IR, incidence rate; MM, multimorbidity, PY, person-years

Supplementary Table 11

**Associations of individual LS7 metrics and sleep duration at baseline with multimorbidity status during follow-up in the Whitehall II and Finnish Public Sector study populations**

|  | **Whitehall II study** | | | **Finnish Public Sector study** | | |
| --- | --- | --- | --- | --- | --- | --- |
| **Metric** | **n MM/N** | **HR** | **95% CI** | **n MM/N** | **HR** | **95% CI** |
| Smoking |  |  |  |  |  |  |
| Non-ideal | 802/2096 | Ref |  | 2812/26851 | Ref |  |
| Ideal | 1949/7619 | 0·55 | 0·51-0·60 | 3663/48526 | 0·71 | 0·68-0·75 |
| Physical activity |  |  |  |  |  |  |
| Non-ideal | 1248/3936 | Ref |  | 2640/25255 | Ref |  |
| Ideal | 1503/5779 | 0·89 | 0·83-0·97 | 3835/50122 | 0·83 | 0·79-0·87 |
| Healthy diet |  |  |  |  |  |  |
| Non-ideal | 2726/9627 | Ref |  | N/A |  |  |
| Ideal | 25/88 | 1·03 | 0·70-1·53 | N/A |  |  |
| Body mass index |  |  |  |  |  |  |
| Non-ideal | 1307/3767 | Ref |  | 3692/33171 | Ref |  |
| Ideal | 1444/5948 | 0·73 | 0·67-0·78 | 2783/42206 | 0·71 | 0·67-0·74 |
| Blood pressure |  |  |  |  |  |  |
| Non-ideal | 1823/6021 | Ref |  | 2015/12933 | Ref |  |
| Ideal | 928/3694 | 0·85 | 0·78-0·92 | 4460/62444 | 0·60 | 0·57-0·63 |
| Total cholesterol |  |  |  |  |  |  |
| Non-ideal | 2215/7314 | Ref |  | 366/2261 | Ref |  |
| Ideal | 536/2401 | 0·87 | 0·79-0·96 | 6109/73116 | 0·77 | 0·69-0·85 |
| FBG |  |  |  |  |  |  |
| Non-ideal | 42/88 | Ref |  | 274/1555 | Ref |  |
| Ideal | 2709/9627 | 0·51 | 0·38-0·70 | 6201/73822 | 0·55 | 0·49-0·62 |
| Sleep duration |  |  |  |  |  |  |
| Non-ideal | N/A |  |  | 2061/19055 | Ref |  |
| Ideal | N/A |  |  | 4369/55871 | 0·82 | 0·77-0·86 |

Hazard ratios and 95% confidence intervals from Cox’s proportional hazards models with age as time scale and birth cohort strata, adjusted for sex, education, occupation, and marital status in Finnish Public Sector study and additionally for ethnicity in Whitehall II study.

Ideal defined according to the criteria in Supplementary Tables 1 and 3.

CI, confidence interval; FBG: fasting blood glucose; HR, hazard ratio; LS7, Life’s Simple 7; N/A: not applicable; MM, multimorbidity

Supplementary Table 12

**Associations of change in individual LS7 metrics and sleep duration from baseline to 1991-1993 (5-year change) in the Whitehall II study population and from baseline to re-assessment (4-year change) in the Finnish Public Sector study population with multimorbidity status during follow-up**

|  |  | **Whitehall II study** | | | | **Finnish Public Sector study** | | | |
| --- | --- | --- | --- | --- | --- | --- | --- | --- | --- |
| **Metric** |  | **Persistently**  **non-ideal** | **Non-ideal →Ideal** | **Ideal→Non-ideal** | **Persistently ideal** | **Persistently**  **non-ideal** | **Non-ideal →Ideal** | **Ideal→Non-ideal** | **Persistently ideal** |
| Smoking | n MM/N | 426/1138 | 83/219 | 131/492 | 1417/5474 | 1298/14751 | 245/2817 | 112/1492 | 2107/33211 |
|  | HR | Ref | 0·90 | 0·63 | 0·57 | Ref | 0·98 | 0·89 | 0·71 |
|  | 95% CI |  | 0·71-1·14 | 0·52-0·77 | 0·51-0·64 |  | 0·86-1·13 | 0·74-1·08 | 0·66-0·76 |
| Physical activity | n MM/N | 630/2010 | 272/868 | 395/1415 | 760/3030 | 956/9836 | 515/7348 | 656/7487 | 1635/27600 |
|  | HR | Ref | 1·01 | 0·92 | 0·87 | Ref | 0·77 | 0·97 | 0·71 |
|  | 95% CI |  | 0·88-1·17 | 0·81-1·05 | 0·78-0·97 |  | 0·69-0·86 | 0·88-1·07 | 0·66-0·77 |
| Healthy diet | n MM/N | 1965/6995 | 68/250 | 18/59 | 6/19 | N/A |  |  |  |
|  | HR | Ref | 1·02 | 1·18 | 1·01 | N/A |  |  |  |
|  | 95% CI |  | 0·80-1·29 | 0·74-1·88 | 0·45-2·26 | N/A |  |  |  |
| Body mass index | n MM/N | 892/2542 | 75/210 | 242/902 | 848/3669 | 1962/20878 | 150/1817 | 303/4463 | 1347/25113 |
|  | HR | Ref | 1·04 | 0·77 | 0·68 | Ref | 0·91 | 0·86 | 0·66 |
|  | 95% CI |  | 0·82-1·32 | 0·67-0·89 | 0·62-0·75 |  | 0·77-1·08 | 0·76-0·97 | 0·62-0·71 |
| Blood pressure | n MM/N | 1129/3535 | 213/938 | 290/1058 | 425/1792 | 1105/8765 | 0 | 420/3997 | 2237/39509 |
|  | HR | Ref | 0·77 | 0·88 | 0·77 | Ref | N/A | 0·92 | 0·57 |
|  | 95% CI |  | 0·66-0·89 | 0·77-1·00 | 0·69-0·86 |  | N/A | 0·82-1·03 | 0·53-0·61 |
| FBG | n MM/N | 20/35 | 2/13 | 470/1480 | 1565/5795 | 145/1033 | 0 | 77/630 | 3540/50608 |
|  | HR | Ref | N/A | 0·52 | 0·46 | Ref | N/A | 0·88 | 0·55 |
|  | 95% CI |  | N/A | 0·33-0·81 | 0·30-0·72 |  | N/A | 0·67-1·16 | 0·47-0·65 |
| Total cholesterol | n MM/N | 1605/5302 | 40/169 | 266/1140 | 146/712 | 183/1556 | 0 | 327/2317 | 3252/48398 |
|  | HR | Ref | 0·94 | 0·89 | 0·85 | Ref | N/A | 1·14 | 0·78 |
|  | 95% CI |  | 0·69-1·29 | 0·78-1·01 | 0·72-1·01 |  | N/A | 0·95-1·37 | 0·67-0·91 |
| Sleep duration | n MM/N | N/A |  |  |  | 742/7535 | 388/5199 | 584/6296 | 2004/32744 |
|  | HR | N/A |  |  |  | Ref | 0·83 | 1·08 | 0·73 |
|  | 95% CI | N/A |  |  |  |  | 0·74-0·94 | 0·97-1·20 | 0·67-0·79 |

Hazard ratios and 95% confidence intervals from Cox’s proportional hazards models with age as time scale and birth cohort strata, adjusted for sex, education, occupation, and marital status in Finnish Public Sector study and additionally for ethnicity in Whitehall II study.

Ideal metric level defined according to criteria in Supplementary Tables 1 and 3.

In the Finnish Public Sector study, blood pressure, fasting blood glucose, and total cholesterol levels were based on medication claims and clinical diagnoses, which explains that change from non-ideal to ideal level is not possible for these metrics: once an individual get diagnosed with diabetes, hypertension, and dyslipidemia, he/she cannot return to ideal level (even if controlled); similarly, drugs for diabetes, hypertension, and dyslipidemia often are lifelong treatments.

CI, confidence interval; FBG, fasting blood glucose; HR, hazard ratio; Life’s Simple 7; N/A, not applicable; MM, multimorbidity

Supplementary Table 13

**Associations of 5-year change in ideal LS7 metrics from baseline to 1991-1993 and 10-year change in LE8 score from baseline to 1997-1999 with multimorbidity status during follow-up in the Whitehall II study population: Sensitivity analysis accounting for cohort attrition using inverse probability weighting as compared to the primary analysis**

|  |  | **IPW analysis** | | **Primary analysis** | |
| --- | --- | --- | --- | --- | --- |
|  | **n MM/N** | **HR** | **95% CI** | **HR** | **95% CI** |
| **5-year-change in ideal LS7 metrics from baseline to 1991-1993** |  |  |  |  |  |
| Change in categories of no. of ideal LS7 metrics |  |  |  |  |  |
| Persistently poor (0-2) | 326/766 | Ref |  | Ref |  |
| Poor (0-2) → Intermediate (3-4) or High (5-7) | 98/280 | 0·75 | 0·63-0·91 | 0·78 | 0·62-0·98 |
| Intermediate (3-4) → Poor (0-2) | 465/1355 | 0·70 | 0·63-0·79 | 0·73 | 0·63-0·84 |
| Persistently intermediate (3-4) | 739/2737 | 0·57 | 0·51-0·64 | 0·59 | 0·52-0·67 |
| Intermediate (3-4) → High (5-7) | 95/412 | 0·49 | 0·40-0·60 | 0·51 | 0·40-0·64 |
| High (5-7) → Poor (0-2) | 39/131 | 0·67 | 0·50-0·90 | 0·70 | 0·50-0·98 |
| High (5-7) → Intermediate (3-4) | 186/989 | 0·42 | 0·36-0·50 | 0·44 | 0·37-0·53 |
| Persistently high (5-7) | 109/653 | 0·40 | 0·33-0·50 | 0·42 | 0·33-0·52 |
| Per one ideal LS7 metric increase* | 2057/7323 | 0·92 | 0·89-0·95 | 0·92 | 0·88-0·96 |
| **10-year change in LE8 score from baseline to 1997-1999** |  |  |  |  |  |
| Change in categories of LE8 score points |  |  |  |  |  |
| Persistently low (0-49) | 23/43 | Ref |  | Ref |  |
| Low (0-49) → Moderate (50-79) or High (80-100) | 23/59 | 0·42 | 0·32-0·54 | 0·49 | 0·28-0·88 |
| Moderate (50-79) → Low (0-49) | 57/165 | 0·52 | 0·41-0·64 | 0·62 | 0·38-1·02 |
| Persistently moderate (50-79) | 578/2008 | 0·33 | 0·28-0·39 | 0·39 | 0·25-0·59 |
| Moderate (50-79) → High (80-100) | 41/185 | 0·22 | 0·17-0·28 | 0·27 | 0·16-0·45 |
| High (80-100) → Moderate (50-79) or Low (0-49) | 106/533 | 0·26 | 0·21-0·33 | 0·32 | 0·21-0·51 |
| Persistently High (80-100) | 48/277 | 0·22 | 0·17-0·28 | 0·27 | 0·16-0·44 |
| Per 10 points LE8 score increase* | 876/3270 | 0·84 | 0·81-0·88 | 0·86 | 0·81-0·93 |

Hazard ratios and 95% confidence intervals from Cox’s proportional hazards models with age as time scale and birth cohort strata, adjusted for sex, ethnicity, education, occupation, and marital status.

The weights correspond to probabilities of attending the examination in 1991-1993/1997-1999 and having complete data on LS7/LE8 (separate analyses) estimated in multivariate logistic regression analysis using baseline characteristics. Adjusting for the inverse of these weights in the Cox’s model accounts for cohort attrition.

*Analysis is further adjusted for baseline ideal LS7 metrics/ baseline LE8 score.

CI, confidence interval; HR, hazard ratio; IPW, inverse probability weighting; LE8, Life’s Essential 8; LS7, Life’s Simple 7; MM, multimorbidity

Supplementary Table 14

**Associations of ideal LS7 metrics at baseline and its 5-year change to 1991-1993 with multimorbidity status during follow-up in the Whitehall II study population: Sensitivity analysis accounting for competing risks as compared to primary analysis**

|  |  | **Competing risk analysis*** | | **Primary analysis** | |
| --- | --- | --- | --- | --- | --- |
|  | **n MM/N** | **subHR** | **95% CI** | **HR** | **95% CI** |
| **Baseline** |  |  |  |  |  |
| No. of ideal LS7 metrics |  |  |  |  |  |
| 0-1 | 130/244 | Ref |  | Ref |  |
| 2 | 522/1350 | 0·66 | 0·54-0·81 | 0·60 | 0·50-0·73 |
| 3 | 953/2954 | 0·56 | 0·46-0·68 | 0·48 | 0·40-0·58 |
| 4 | 745/2961 | 0·46 | 0·38-0·56 | 0·39 | 0·32-0·47 |
| 5 | 325/1737 | 0·36 | 0·20-0·45 | 0·30 | 0·25-0·37 |
| 6-7 | 76/469 | 0·35 | 0·26-0·47 | 0·30 | 0·22-0·40 |
| Per one ideal LS7 metric | 2751/9715 | 0·82 | 0·79-0·85 | 0·79 | 0·76-0·82 |
| **5-year change from baseline to 1991-1993** |  |  |  |  |  |
| Change in categories of no. of ideal LS7 metrics |  |  |  |  |  |
| Persistently poor (0-2) | 326/766 | Ref |  | Ref |  |
| Poor (0-2) → Intermediate (3-4) or High (5-7) | 98/280 | 0·81 | 0·65-1·03 | 0·78 | 0·62-0·98 |
| Intermediate (3-4) → Poor (0-2) | 465/1355 | 0·79 | 0·68-0·91 | 0·73 | 0·63-0·84 |
| Persistently intermediate (3-4) | 739/2737 | 0·65 | 0·57-0·75 | 0·59 | 0·52-0·67 |
| Intermediate (3-4) → High (5-7) | 95/412 | 0·59 | 0·47-0·74 | 0·51 | 0·40-0·64 |
| High (5-7) → Poor (0-2) | 39/131 | 0·79 | 0·56-1·12 | 0·70 | 0·50-0·98 |
| High (5-7) → Intermediate (3-4) | 186/989 | 0·49 | 0·41-0·59 | 0·44 | 0·37-0·53 |
| Persistently high (5-7) | 109/653 | 0·48 | 0·38-0·59 | 0·42 | 0·33-0·52 |
| Per one ideal LS7 metric increase^†^ | 2057/7323 | 0·93 | 0·89-0·97 | 0·92 | 0·88-0·96 |

Hazard ratios and 95% confidence intervals from Fine and Gray sub-distribution hazard models with age as time scale and birth cohort strata, adjusted for sex, ethnicity, education, occupation, and marital status.

*Compering risk analysis was conducted using Fine and Grey method, and sub distribution hazard ratios were estimated. ^3^

^†^Analysis is further adjusted for baseline ideal LS7 metrics.

CI, confidence interval; HR, hazard ratio; LS7, Life’s Simple 7; MM, multimorbidity; subHR, sub-distribution hazard ratio

Supplementary Table 15

**Associations of LE8 score at baseline and its 10-year change to 1997-1999 with multimorbidity status during follow-up in the Whitehall II study population: Sensitivity analysis accounting for competing risks as compared to primary analysis**

|  |  | **Competing risk analysis*** | | **Primary analysis** | |
| --- | --- | --- | --- | --- | --- |
|  | **n MM/N** | **subHR** | **95% CI** | **HR** | **95% CI** |
| **Baseline** |  |  |  |  |  |
| Categories of LE8 score points |  |  |  |  |  |
| Low (0-49) | 254/549 | Ref |  | Ref |  |
| Moderate (50-80) | 2138/7177 | 0·70 | 0·61-0·80 | 0·58 | 0·50-0·66 |
| High (80-100) | 368/2003 | 0·50 | 0·42-0·59 | 0·41 | 0·35-0·48 |
| Per 10 points LE8 score | 2760/9729 | 0·80 | 0·78-0·83 | 0·77 | 0·74-0·79 |
| **10-year change from baseline to 1997-1999** |  |  |  |  |  |
| Change in categories of LE8 score points |  |  |  |  |  |
| Persistently low (0-49) | 23/43 | Ref |  | Ref |  |
| Low (0-49) → Moderate (50-79) or High (80-100) | 23/59 | 0·50 | 0·28-0·90 | 0·49 | 0·28-0·88 |
| Moderate (50-79) → Low (0-49) | 57/165 | 0·66 | 0·40-1·09 | 0·62 | 0·38-1·02 |
| Persistently Moderate (50-79) | 578/2008 | 0·44 | 0·29-0·68 | 0·39 | 0·25-0·59 |
| Moderate (50-79) → High (80-100) | 41/185 | 0·32 | 0·19-0·53 | 0·27 | 0·16-0·45 |
| High (80-100) → Moderate (50-79) or Low (0-49) | 106/533 | 0·37 | 0·23-0·59 | 0·32 | 0·21-0·51 |
| Persistently High (80-100) | 48/277 | 0·31 | 0·19-0·52 | 0·27 | 0·16-0·44 |
| Per 10 points LE8 score increase^†^ | 876/3270 | 0·89 | 0·83-0·95 | 0·86 | 0·81-0·93 |

Hazard ratios and 95% confidence intervals from Cox’s proportional hazards models with age as time scale and birth cohort strata, adjusted for sex, ethnicity, education, occupation, and marital status.

*Compering risk analysis was conducted using Fine and Grey method, and sub distribution hazard ratios were estimated. ^3^

^†^Analysis is further adjusted for baseline LE8 score.

CI, confidence interval; HR, hazard ratio; LE8, Life’s Essential 8; MM, multimorbidity; subHR, sub-distribution hazard ratio

Supplementary Table 16

**Associations of ideal LS7 metrics at baseline and its 5-year change to 1991-1993 with multimorbidity status during follow-up in the Whitehall II study population: Sensitivity analyses excluding single chronic disease at baseline and re-assessment and censoring after 15 years**

|  | **Excluding singly chronic diseases** | | | **Censoring after 15 years** | | |
| --- | --- | --- | --- | --- | --- | --- |
|  | **n MM/N** | **HR** | **95% CI** | **n MM/N** | **HR** | **95% CI** |
| **Baseline** |  |  |  |  |  |  |
| No. of ideal LS7 metrics |  |  |  |  |  |  |
| 0-1 | 126/239 | Ref |  | 17/244 | Ref |  |
| 2 | 511/1317 | 0·61 | 0·50-0·75 | 67/1350 | 0·75 | 0·44-1·28 |
| 3 | 932/2889 | 0·49 | 0·41-0·59 | 77/2954 | 0·44 | 0·26-0·75 |
| 4 | 730/2916 | 0·39 | 0·32-0·48 | 59/2961 | 0·38 | 0·22-0·66 |
| 5 | 315/1708 | 0·30 | 0·25-0·38 | 21/1737 | 0·26 | 0·14-0·50 |
| 6-7 | 73/460 | 0·30 | 0·22-0·40 | 7/469 | 0·37 | 0·15-0·91 |
| Per one ideal LS7 metric | 2687/9529 | 0·79 | 0·76-0·82 | 248/9715 | 0·74 | 0·66-0·83 |
| **5-year-change from baseline to 1991-1993** |  |  |  |  |  |  |
| Change in categories of no. of ideal LS7 metrics |  |  |  |  |  |  |
| Persistently poor (0-2) | 320/738 | Ref |  | 98/766 | Ref |  |
| Poor (0-2) → Intermediate (3-4) or High (5-7) | 97/268 | 0·80 | 0·63-1·00 | 30/280 | 0·84 | 0·56-1·27 |
| Intermediate (3-4) → Poor (0-2) | 460/1308 | 0·73 | 0·63-0·84 | 120/1355 | 0·72 | 0·55-0·94 |
| Persistently intermediate (3-4) | 723/2656 | 0·59 | 0·51-0·67 | 157/2737 | 0·50 | 0·39-0·65 |
| Intermediate (3-4) → High (5-7) | 92/402 | 0·50 | 0·39-0·63 | 12/412 | 0·27 | 0·15-0·49 |
| High (5-7) → Poor (0-2) | 39/128 | 0·71 | 0·51-1·00 | 9/131 | 0·64 | 0·32-1·27 |
| High (5-7) → Intermediate (3-4) | 180/960 | 0·43 | 0·36-0·52 | 33/989 | 0·33 | 0·22-0·50 |
| Persistently high (5-7) | 106/636 | 0·41 | 0·33-0·52 | 18/653 | 0·30 | 0·18-0·51 |
| Per one ideal LS7 metric increase* | 2017/7096 | 0·92 | 0·88-0·96 | 477/7323 | 0·84 | 0·77-0·92 |

Hazard ratios and 95% confidence intervals from Cox’s proportional hazards models with age as time scale and birth cohort strata, adjusted for sex, ethnicity, education, occupation, and marital status.

*Analysis is further adjusted for baseline ideal LS7 metrics.

CI, confidence interval; HR, hazard ratio; LS7, Life’s Simple 7; MM, multimorbidity.

Supplementary Table 17

**Associations of LE8 score at baseline and its 10-year change to 1997-1999 with multimorbidity status during follow-up in the Whitehall II study population: Sensitivity analyses excluding single chronic disease at baseline and re-assessment and censoring after 15 years**

|  | **Excluding singly chronic diseases** | | | **Censoring after 15 years** | | |
| --- | --- | --- | --- | --- | --- | --- |
|  | **n MM/N** | **HR** | **95% CI** | **n MM/N** | **HR** | **95% CI** |
| **Baseline** |  |  |  |  |  |  |
| Categories of LE8 score points |  |  |  |  |  |  |
| Low (0-49) | 249/533 | Ref |  | 32/549 | Ref |  |
| Moderate (50-80) | 2089/7035 | 0·57 | 0·50-0·65 | 190/7177 | 0·60 | 0·41-0·88 |
| High (80-100) | 358/1976 | 0·40 | 0·34-0·47 | 26/2003 | 0·39 | 0·23-0·67 |
| Per 10 points LE8 score | 2696/9544 | 0·76 | 0·74-0·79 | 248/9729 | 0·76 | 0·68-0·85 |
| **Change from baseline to 1997-1999** |  |  |  |  |  |  |
| Change in categories of LE8 score points |  |  |  |  |  |  |
| Persistently low (0-49) | 23/38 | Ref |  | 6/43 | Ref |  |
| Low (0-49) → Moderate (50-79) or High (80-100) | 23/55 | 0·46 | 0·26-0·81 | 4/59 | 0·37 | 0·11-1·33 |
| Moderate (50-79) → Low (0-49) | 57/156 | 0·57 | 0·35-0·93 | 15/165 | 0·86 | 0·33-2·23 |
| Persistently Moderate (50-79) | 571/1902 | 0·35 | 0·23-0·53 | 123/2008 | 0·47 | 0·21-1·09 |
| Moderate (50-79) → High (80-100) | 40/179 | 0·23 | 0·14-0·38 | 8/185 | 0·31 | 0·11-0·90 |
| High (80-100) → Moderate (50-79) or Low (0-49) | 103/507 | 0·28 | 0·18-0·45 | 19/533 | 0·34 | 0·13-0·86 |
| Persistently High (80-100) | 47/265 | 0·23 | 0·14-0·38 | 6/277 | 0·21 | 0·07-0·65 |
| Per 10 points LE8 score increase* | 864/3102 | 0·86 | 0·80-0·92 | 181/3270 | 0·80 | 0·68-0·93 |

Hazard ratios and 95% confidence intervals from Cox’s proportional hazards models with age as time scale and birth cohort strata, adjusted for sex, ethnicity, education, occupation, and marital status.

*Analysis is further adjusted for baseline LE8 score.

CI, confidence interval; HR, hazard ratio; LE8, Life’s Essential 8; MM, multimorbidity

Supplementary Table 18

**Associations of ideal LS7 metrics at baseline and its 5-year change to 1991-1993 with multimorbidity status during follow-up in the Whitehall II study: Sensitivity analysis excluding cardiovascular disease from multimorbidity**

|  | **n MM/N** | **HR** | **95% CI** |
| --- | --- | --- | --- |
| **Baseline** |  |  |  |
| No. of ideal LS7 metrics |  |  |  |
| 0-1 | 37/151 | Ref |  |
| 2 | 191/1019 | 0·63 | 0·45-0·90 |
| 3 | 335/2336 | 0·47 | 0·33-0·66 |
| 4 | 304/2520 | 0·42 | 0·30-0·59 |
| 5 | 145/1557 | 0·34 | 0·23-0·49 |
| 6-7 | 40/433 | 0·38 | 0·24-0·60 |
| Per one ideal LS7 metric | 1052/8016 | 0·83 | 0·78-0·87 |
| **5-year change from baseline to 1991-1993** |  |  |  |
| Change in categories of no. of ideal LS7 metrics |  |  |  |
| Persistently poor (0-2) | 108/548 | Ref |  |
| Poor (0-2) → Intermediate (3-4) or High (5-7) | 33/215 | 0·77 | 0·52-1·14 |
| Intermediate (3-4) → Poor (0-2) | 166/1056 | 0·73 | 0·57-0·93 |
| Persistently intermediate (3-4) | 285/2283 | 0·60 | 0·48-0·75 |
| Intermediate (3-4) → High (5-7) | 45/362 | 0·60 | 0·42-0·85 |
| High (5-7) → Poor (0-2) | 18/110 | 0·86 | 0·52-1·42 |
| High (5-7) → Intermediate (3-4) | 80/883 | 0·47 | 0·35-0·63 |
| Persistently high (5-7) | 56/600 | 0·51 | 0·37-0·71 |
| Per one ideal LS7 metric increase* | 791/6057 | 0·93 | 0·87-1·00 |

Hazard ratios and 95% confidence intervals from Cox’s proportional hazards models with age as time scale and birth cohort strata, adjusted for sex, ethnicity, education, occupation, and marital status.

*Analysis is further adjusted for baseline ideal LS7 metrics.

CI, confidence interval; CVD, cardiovascular disease; HR, hazard ratio; LS7, Life’s Simple 7; MM, multimorbidity

Supplementary Table 19

**Associations of LE8 score at baseline and its 10-year change to 1997-1999 with multimorbidity status during follow-up in the Whitehall II study population: Sensitivity analysis excluding cardiovascular disease from multimorbidity**

|  | **n MM/N** | **HR** | **95% CI** |
| --- | --- | --- | --- |
| **Baseline** |  |  |  |
| Categories of LS8 score points |  |  |  |
| Low (0-49) | 81/376 | Ref |  |
| Moderate (50-80) | 814/5853 | 0·56 | 0·45-0·71 |
| High (80-100) | 158/1793 | 0·41 | 0·31-0·54 |
| Per 10 points LE8 score | 1053/8022 | 0·78 | 0·74-0·83 |
| **10-year change from baseline to 1997-1999** |  |  |  |
| Change in categories of LE8 score points |  |  |  |
| Persistently low (0-49) | 7/27 | Ref |  |
| Low (0-49) → Moderate (50-79) or High (80-100) | 9/45 | 0·64 | 0·24-1·72 |
| Moderate (50-79) → Low (0-49) | 19/127 | 0·57 | 0·24-1·36 |
| Persistently Moderate (50-79) | 229/1659 | 0·43 | 0·20-0·91 |
| Moderate (50-79) → High (80-100) | 16/160 | 0·26 | 0·11-0·65 |
| High (80-100) → Moderate (50-79) or Low (0-49) | 37/464 | 0·30 | 0·13-0·69 |
| Persistently High (80-100) | 25/254 | 0·34 | 0·15-0·80 |
| Per 10 points LE8 score increase* | 342/2736 | 0·97 | 0·87-1·09 |

Hazard ratios and 95% confidence intervals from Cox’s proportional hazards models with age as time scale and birth cohort strata, adjusted for sex, ethnicity, education, occupation, and marital status.

*Analysis is further adjusted for baseline LE8 score.

CI, confidence interval; HR, hazard ratio; LE8, Life’s Essential 8; MM, multimorbidity

Supplementary Table 20

**Associations of ideal LS7 metrics at baseline and its 5-year change to 1991-1993 with multimorbidity status during follow-up in the Whitehall II study: Sensitivity analysis further adjusted for post-hoc covariates**

|  | **n MM/N** | **HR** | **95% CI** |
| --- | --- | --- | --- |
| **Baseline** |  |  |  |
| No. of ideal LS7 metrics |  |  |  |
| 0-1 | 112/212 | Ref |  |
| 2 | 473/1243 | 0.59 | 0.48-0.73 |
| 3 | 865/2685 | 0.50 | 0.41-0.61 |
| 4 | 697/2723 | 0.41 | 0.33-0.50 |
| 5 | 293/1597 | 0.31 | 0.25-0.39 |
| 6-7 | 67/440 | 0.30 | 0.22-0.41 |
| Per one ideal LS7 metric | 2507/8900 | 0.80 | 0.77-0.83 |
| **5-year change from baseline to 1991-1993** |  |  |  |
| Change in categories of no. of ideal LS7 metrics |  |  |  |
| Persistently poor (0-2) | 298/708 | Ref |  |
| Poor (0-2) → Intermediate (3-4) or High (5-7) | 86/259 | 0.74 | 0.58-0.95 |
| Intermediate (3-4) → Poor (0-2) | 427/1234 | 0.76 | 0.65-0.88 |
| Persistently intermediate (3-4) | 691/2531 | 0.62 | 0.54-0.71 |
| Intermediate (3-4) → High (5-7) | 90/393 | 0.53 | 0.42-0.68 |
| High (5-7) → Poor (0-2) | 33/119 | 0.68 | 0.47-0.97 |
| High (5-7) → Intermediate (3-4) | 170/916 | 0.47 | 0.39-0.57 |
| Persistently high (5-7) | 96/608 | 0.42 | 0.33-0.53 |
| Per one ideal LS7 metric increase* | 1891/6768 | 0.92 | 0.88-0.96 |

Hazard ratios and 95% confidence intervals from Cox’s proportional hazards models with age as time scale and birth cohort strata, adjusted for sex, ethnicity, education, occupation, marital status *plus* family history of angina, myocardial infarction, stroke, diabetes, and cancer death, Townsend score, and alcohol consumption.

*Analysis is further adjusted for baseline ideal LS7 metrics.

CI, confidence interval; HR, hazard ratio; LS7, Life’s Simple 7; MM, multimorbidity

Supplementary Table 21

**Associations of LE8 score at baseline and its 10-year change to 1997-1999 with multimorbidity status during follow-up in the Whitehall II study population: Sensitivity analysis further adjusted for post-hoc covariates**

|  | **n MM/N** | **HR** | **95% CI** |
| --- | --- | --- | --- |
| **Baseline** |  |  |  |
| Categories of LS8 score points |  |  |  |
| Low (0-49) | 230/508 | Ref |  |
| Moderate (50-80) | 1951/6549 | 0.61 | 0.53-0.70 |
| High (80-100) | 335/1859 | 0.43 | 0.36-0.51 |
| Per 10 points LE8 score | 2516/8916 | 0.77 | 0.75-0.80 |
| **10-year change from baseline to 1997-1999** |  |  |  |
| Change in categories of LE8 score points |  |  |  |
| Persistently low (0-49) | 21/41 | Ref |  |
| Low (0-49) → Moderate (50-79) or High (80-100) | 21/56 | 0.53 | 0.29-0.97 |
| Moderate (50-79) → Low (0-49) | 51/152 | 0.70 | 0.42-1.17 |
| Persistently Moderate (50-79) | 539/1864 | 0.44 | 0.28-0.69 |
| Moderate (50-79) → High (80-100) | 39/175 | 0.32 | 0.19-0.55 |
| High (80-100) → Moderate (50-79) or Low (0-49) | 97/496 | 0.39 | 0.24-0.62 |
| Persistently High (80-100) | 45/263 | 0.31 | 0.18-0.53 |
| Per 10 points LE8 score increase* | 813/3047 | 0.88 | 0.81-0.94 |

Hazard ratios and 95% confidence intervals from Cox’s proportional hazards models with age as time scale and birth cohort strata, adjusted for sex, ethnicity, education, occupation, marital status *plus* family history of angina, myocardial infarction, stroke, diabetes, and cancer death, Townsend score, and alcohol consumption.

*Analysis is further adjusted for baseline LE8 score.

CI, confidence interval; HR, hazard ratio; LE8, Life’s Essential 8; MM, multimorbidity

Supplementary Figure 1

**Study design for the primary analysis in the Whitehall II study. Analyses of baseline and change in LS7 metrics and score (Part A) and of baseline and change in LE8 score (Part B)**

**
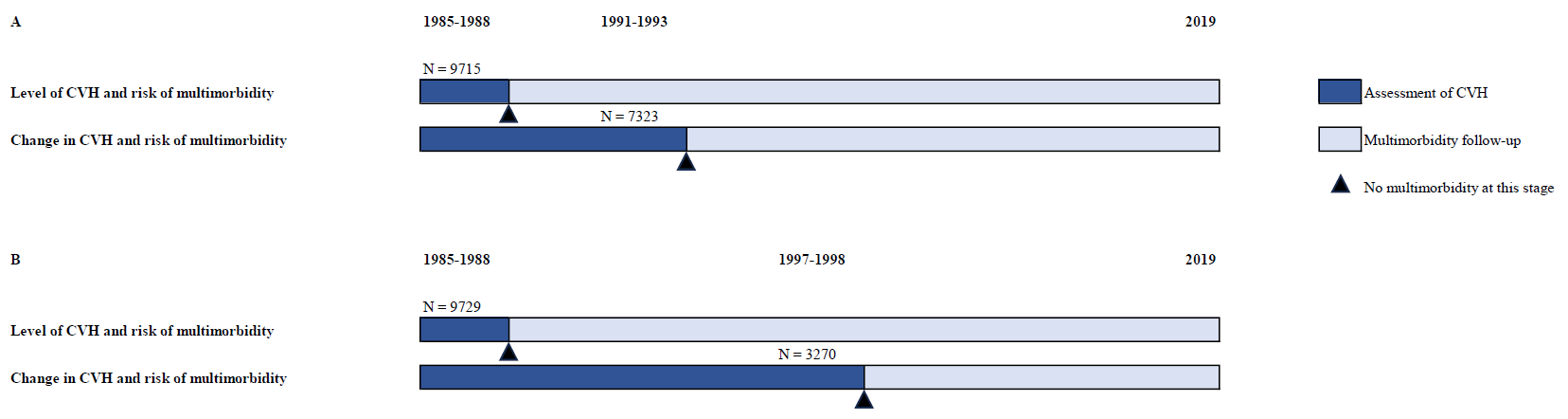
**

**Supplementary Figure 2**

**Study design for the validation analysis in the Finnish Public Sector study: Analyses of baseline and change in LS7 metrics**


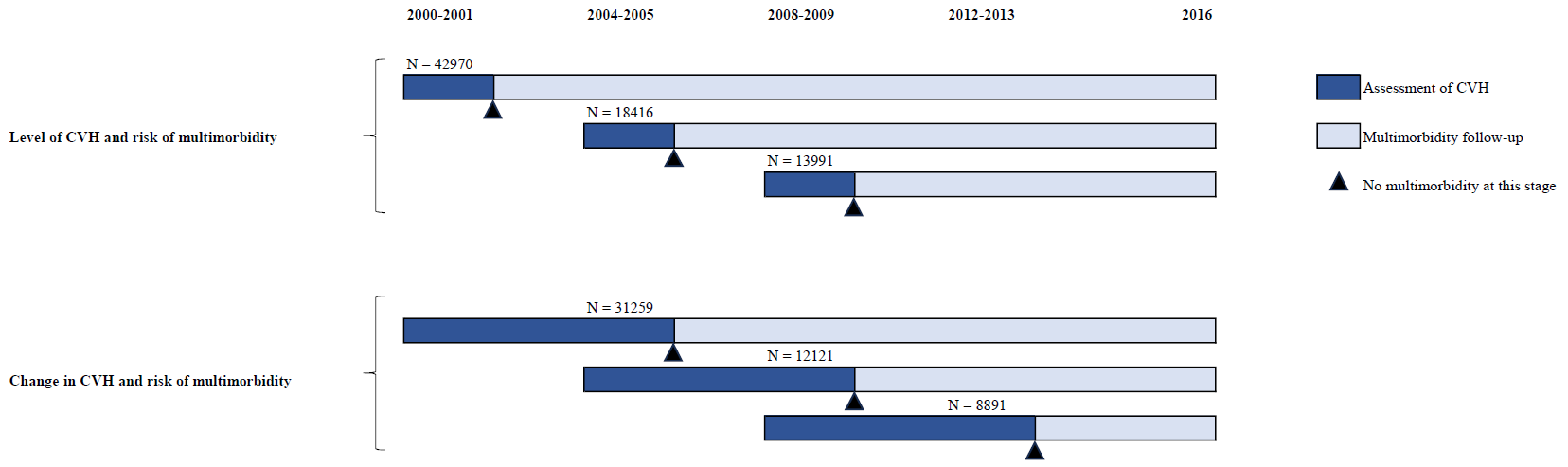


Supplementary Figure 3

**Flow chart showing numbers of participants included and excluded from the eligible study population in the Whitehall II study. Analyses of baseline and change in LS7 metrics and score (Part A) and of baseline and change in LE8 score (Part B)**

**A**

| *Eligible study population:*  N=10308 at baseline (1985-1988) | |  |  |
| --- | --- | --- | --- |
|  |  |  | N=3 with history of MM |
|  |  |  |  |
| N=10305 without history of MM | |  |  |
|  |  |  | N=9 without link to NHS electronic health records |
|  |  |  |  |
| N=10296 with link to NHS electronic health records | |  |  |
|  |  |  | N=353 without LS7 data |
|  |  |  |  |
| N=9943 with LS7 data | |  |  |
|  |  |  | N=116 with history of CVD |
|  |  |  |  |
| N=9827 without history of CVD | |  | |
|  |  |  | N=112 with missing covariates |
|  |  |  |  |
| *Analysis of baseline LS7:*  N=9715 without missing covariates at baseline; Median follow-up: 31·4 years; MM events: N=2751 | |  |  |

| N=9715 without missing covariates at baseline | |  |  |
| --- | --- | --- | --- |
|  |  |  |  |
|  |  |  | N=103 died in the interval (i.e. before examination in 1991-1993) |
|  |  |  |  |
| N=9612 survived the interval | |  |  |
|  |  |  |  |
|  |  |  | N=1241 not attending examination in 1991-1993 |
|  |  |  |  |
| N= 8371 attended examination in 1991-1993 | |  |  |
|  |  |  |  |
|  |  |  | N=11 with prevalent MM at examination in 1991-1993 |
|  |  |  |  |
| N=8360 without prevalent MM at examination in 1991-1993 | |  |  |
|  |  |  |  |
|  |  |  | N=1037 with missing LS7 data at examination in 1991-1993 |
|  |  |  |  |
| *Analysis of change in LS7:*  N=7323 with LS7 data at examination in 1991-1993; Median follow-up: 26·5 years; MM events: N=2057 | |  |  |
|  |  |  |  |

**B**

| *Eligible study population:*  N=10308 at baseline (1985-1988) | |  |  |
| --- | --- | --- | --- |
|  |  |  | N=3 with history of MM |
|  |  |  |  |
| N=10305 without history of MM | |  |  |
|  |  |  | N=9 without link to NHS electronic health records |
|  |  |  |  |
| N=10296 with link to NHS electronic health records | |  |  |
|  |  |  | N=342 without LS8 data |
|  |  |  |  |
| N=9954 with LS8 data | |  |  |
|  |  |  | N=113 with history of CVD |
|  |  |  |  |
| N=9841 without history of CVD | |  | |
|  |  |  | N=112 with missing covariates |
|  |  |  |  |
| *Analysis of baseline LE8:*  N=9729 without missing covariates at baseline; Median follow-up: 31·4 years; MM events: N=2760 | |  |  |

| N=9729 without missing covariates at baseline | |  |  |
| --- | --- | --- | --- |
|  |  |  |  |
|  |  |  | N=262 died in the interval (i.e. before examination in 1997-1999) |
|  |  |  |  |
| N=9467 survived the interval | |  |  |
|  |  |  |  |
|  |  |  | N=1955 not attending examination in 1997-1999 |
|  |  |  |  |
| N=7512 attending examination in 1997-1999 | |  |  |
|  |  |  |  |
|  |  |  | N=60 with prevalent MM at examination in 1997-1999 |
|  |  |  |  |
| N=7452 without prevalent MM at examination in 1997-1999 | |  |  |
|  |  |  |  |
|  |  |  | N=4182 with missing LE8 data at examination in 1997-1999 |
|  |  |  |  |
| *Analysis of change in LE8:*  N=3270 with LE8 data at examination in 1997-1999; Median follow-up: 20·8 years; MM events: N=876 | |  |  |
|  |  |  |  |

LE8, Life’s Essential 8; LS7, Life’s Simple 7; MM, multimorbidity; CVD, cardiovascular disease

Supplementary Figure 4

**Flow chart showing number of participants included and excluded from the eligible study population in the Finnish Public Sector study: Analyses of baseline and change in LS7 metrics**

| *Eligible study population:*  N=83581 in at least one survey (2000-02, 2004-05, 2008-09) | |  |  |
| --- | --- | --- | --- |
|  |  |  | N=5356 with missing LS7 data |
|  |  |  |  |
| N=78225 without missing LS7 data | |  |  |
|  |  |  | N=685 with prevalent CVD |
|  |  |  |  |
| N=77540 without history of CVD | |  |  |
|  |  |  | N=849 with prevalent MM |
|  |  |  |  |
| N=76691 without prevalent MM | |  |  |
|  |  |  | N=1314 with missing covariates |
|  |  |  |  |
| *Analysis of baseline LS7:*  N=75377 without missing covariates at baseline; Median follow-up: 14·7 years; MM events: N=6475 | |  |  |

| N=75377 without missing covariates at baseline | |  |  |
| --- | --- | --- | --- |
|  |  |  |  |
|  |  |  | N=281 died in the interval (i.e. before second evaluation) |
|  |  |  |  |
| N=75096 survived the interval | |  |  |
|  |  |  |  |
|  |  |  | N=19863 not attending second evaluation (2004-05, 2008-2009, 2012-2013) |
|  |  |  |  |
| N=55233 attended second evaluation (2004-2005, 2008-2009, 2012-2013) | |  |  |
|  |  |  |  |
|  |  |  | N=799 with prevalent MM at second evaluation |
|  |  |  |  |
| N=54434 without prevalent MM at the second evaluation | |  |  |
|  |  |  |  |
|  |  |  | N=2163 with missing LS7 data at second evaluation |
|  |  |  |  |
| *Analysis of change in LS7:*  N=52271 with LS7 data at re-assessment; Median follow-up: 11·6 years; MM events: N=3762 | |  |  |
|  |  |  |  |

LS7, Life’s Simple 7; MM, multimorbidity; CVD, cardiovascular disease

Data access application

Analysis plan for replication

Study protocol

Statistical code

Whitehall II study / Statistical code (SAS and R):

**proc** **format**;

value nbcvhgroup

**0**-**1** = '0-1'

**2** = '2'

**3** = '3'

**4** = '4'

**5** = '5'

**6**-**7** = "6-7"

;

**run**;

**proc** **format**;

value $combigp

'01' = '0.'

'02' = '0.'

;

**run**;

**************************************************************************

*** Table 1 ***

**************************************************************************;

**proc** **freq** data=base7;

table sex ethn_ds edlevr grlump statusx coh nbid;

**run**;

**proc** **means** data=base7 n mean std maxdec=**1**;

var age_c nbid;**run**;

**************************************************************************

*** Table 2 ***

**************************************************************************;

**proc** **freq** data=base7;format nbid nbcvhgroup.;

table nbid*multmorb;

**run**;

*Incidence rate (lndmm=log(delmultmorb));**proc** **genmod** data= base7;

model multmorb= /dist=poi link=log offset= lndmm;

estimate "log(rate)" intercept **1**;

**run**;

**proc** **genmod** data=base7;

class nbid;

model multmorb = nbid / dist=poisson offset=lndmm;

lsmeans nbid / exp cl;

format nbid nbcvhgroup.;

**run**;

**proc** **phreg** data=base7;

strata coh;

class sex ethn_ds edlevr grlump statusx;

model (age_c,age_mm)*multmorb(**0**)=nbid sex ethn_ds edlevr grlump statusx/rl;

**run**;

**proc** **phreg** data=base7;format nbid nbcvhgroup.;

strata coh;

class nbid(ref='0-1') sex ethn_ds edlevr grlump statusx;

model (age_c,age_mm)*multmorb(**0**)=nbid sex ethn_ds edlevr grlump statusx/rl;

**run**;

**************************************************************************

*** Table 3 ***

**************************************************************************;

**proc** **format**;

value cvh8group

LOW -<**50** = '0 Low'

**50**-<**80** = '1 Moderate'

**80** - HIGH = '2 High'

;

**run**;

**proc** **format**;

value $combiscoregp

'0 Low1 Moderate' = '0 Low Mod/High'

'0 Low2 High' = '0 Low Mod/High'

'2 High0 Low' = '2 High Low/Mod'

'2 High1 Moderate' = '2 High Low/Mod'

;

**run**;

**proc** **freq** data=base8;format scoret0_cvh8 cvh8group.;

table scoret0_cvh8*multmorb;

**run**;

**proc** **genmod** data= base8;

model multmorb= /dist=poi link=log offset= lndmm;

estimate "log(rate)" intercept **1**;

**run**;

**proc** **genmod** data=base8;

class scoret0_cvh8;

model multmorb = scoret0_cvh8 / dist=poisson offset=lndmm;

lsmeans scoret0_cvh8 / exp cl;

format scoret0_cvh8 cvh8group.;

**run**;

**proc** **phreg** data=base8;

strata coh;

class sex ethn_ds edlevr grlump statusx;

model (age_c,age_mm)*multmorb(**0**)=scoret0_cvh8 sex ethn_ds edlevr grlump statusx/rl;

hazardratio scoret0_cvh8/units=**10**;

**run**;

**proc** **phreg** data=base8;format scoret0_cvh8 cvh8group.;

strata coh;

class scoret0_cvh8(ref='0 Low') sex ethn_ds edlevr grlump statusx;

model (age_c,age_mm)*multmorb(**0**)=scoret0_cvh8 sex ethn_ds edlevr grlump statusx/rl;

**run**;

**proc** **freq** data=change8;format combiscore $combiscoregp.;

table combiscore*multmorb;

**run**;

**proc** **genmod** data= change8;

model multmorb= /dist=poi link=log offset= lndmm5;

estimate "log(rate)" intercept **1**;

**run**;

**proc** **genmod** data=change8;

class combiscore;

model multmorb = combiscore / dist=poisson offset=lndmm5;

lsmeans combiscore / exp cl;

format combiscore $combiscoregp.;

**run**;

**proc** **phreg** data=change8;

strata coh;

class sex ethn_ds edlevr grlump statusx;

model (tage_c,age_mm)*multmorb(**0**)=deltscor scoret0_cvh8 sex ethn_ds edlevr grlump statusx/rl;

hazardratio deltscor/units=**10**;

**run**;

**proc** **phreg** data=change8;format combiscore $combiscoregp.;

strata coh;

class sex ethn_ds edlevr grlump statusx;

class combiscore(ref='0 Low0 Low');

model (tage_c,age_mm)*multmorb(**0**)=combiscore sex ethn_ds edlevr grlump statusx/rl;

**run**;

**************************************************************************

*** Figure 1A ***

**************************************************************************;

**proc** **format**;

value $combigpgraph

'00' = "P - P"

'01' = "P - I/H"

'02' = "P - I/H"

'10' = "I - P"

'11' = "I - I"

'12' = "I - H"

'20' = "H - P"

'21' = "H - I"

'22' = 'H - H'

;

**run**;

ods graphics on;

ods output Survivalplot=SurvivalPlotData;

ods graphics/reset=all;

**proc** **lifetest** data=change7 plots=s (atrisk=**0** to **30** by **5**);format combi $combigpgraph. ;

time delmmchgt3*multmorb(**0**);strata combi;

**run**;

**data** SurvPlotbase;set SurvivalPlotData;

inc=(**1**-Survival)***100**;

**run**;

ods listing image_dpi=**300** ;

ods graphics on / outputfmt=tiff antialiasmax=**10000**;

**proc** **format**;

value $combigpgraphor (NOTSORTED)

"P - P"="P (*ESC*){Unicode '2192'x} P"

"P - I/H"="P (*ESC*){Unicode '2192'x} I/H"

"I - P"="I (*ESC*){Unicode '2192'x} P"

"I - I"="I (*ESC*){Unicode '2192'x} I"

"I - H"="I (*ESC*){Unicode '2192'x} H"

"H - P"="H (*ESC*){Unicode '2192'x} P"

"H - I"="H (*ESC*){Unicode '2192'x} I"

"H - H"="H (*ESC*){Unicode '2192'x} H"

;

**run**;

**data** label_at_risk;

function='text'; x1space='graphpercent'; y1space='graphpercent';

textsize=**10**; textweight='normal';textfont='calibri';y1=**33**;x1=**6**;label='Change';

**run**;

**proc** **sgplot** data=SurvPlotbase noborder noautolegend sganno=label_at_risk;where time<**27**;

styleattrs DATACONTRASTCOLORS=("CXA5A5A5" "CXA5A5A5" "CXA5A5A5" "CX5B9BD5" "CX5B9BD5" "CX5B9BD5" "CXED7D31" "CXED7D31")

DATALINEPATTERNS=(**1** **5** **2** **5** **1** **2** **2** **1**);

step x=time y=inc / group=stratum name='s' curvelabel curvelabelpos =end grouporder=data curvelabelattrs=(family="calibri" size=**11**pt) lineattrs=(thickness=**1.5** pt);

xaxistable atrisk / x=tatrisk class=stratum colorgroup=stratum valueattrs=(family="calibri" size=**10**pt ) title="Number at risk" titleattrs=(family="calibri" size=**10**pt) classorder=descending

;

xaxis label='Follow-up (years)' labelattrs=(family="calibri" size=**10**pt) valueattrs=(family="calibri" size=**10**pt) values=(**0** to **25** by **5**);

yaxis label='Incidence of multimorbidity (%)' labelattrs=(family="calibri" size=**10**pt size=**10**pt) valueattrs=(family="calibri" size=**10**pt) ;*values=(0 to 0.25 by 0.05);

format stratum $combigpgraphor.;

**run**;

ods graphics off;

**************************************************************************

*** Figure 2A , 2B and Primary analysis of change in Supplementary Tables 13 and 14 ***

**************************************************************************;

**proc** **freq** data=change7;format combi $combigp.;

table combi*multmorb;

**run**;

**proc** **genmod** data= change7;

model multmorb= /dist=poi link=log offset= lndmm;

estimate "log(rate)" intercept **1**;

**run**;

**proc** **genmod** data= change7;

class combi;

model multmorb = combi / dist=poisson offset=lndmm;

lsmeans combiscore / exp cl;

format combiscore $ combigp.;

**run**;

**proc** **phreg** data=change7;

strata coh;

class sex ethn_ds edlevr grlump statusx;

model (xage_c,age_mm)*multmorb(**0**)= deltnbid nbid sex ethn_ds edlevr grlump statusx/rl;

**run**;

**proc** **phreg** data=change7;format combi $combigp. ;

strata coh ;

class combi(ref='00') sex ethn_ds edlevr grlump statusx;

model (xage_c,age_mm)*multmorb(**0**)=combi sex ethn_ds edlevr grlump statusx/rl;

**run**;

* R code for the graph;

library(forestplot)

library(extrafont)

loadfonts(device="win")

fonts()

tabletext<-cbind(c("","Per one ideal LS7 metric increase","Change in categories of no. of ideal LS7 metrics"," Persistently poor (0-2)"," Poor (0-2) ? Intermediate (3-4) or High (5-7)"," Intermediate (3-4) ? Poor (0-2)"," Persistently intermediate (3-4)"," Intermediate (3-4) ? High (5-7)"," High (5-7) ? Poor (0-2) "," High (5-7) ? Intermediate (3-4)"," Persistently high (5-7)"),

c("n MM/N","2057/7323",NA,"326/766","98/280","465/1355","739/2737","95/412","39/131","186/989","109/653"),

c("IR per 1000 PY","",NA,"19.9","15.8","14.9","11.3","9.2","12.6","7.6","6.6"),

c("HR (95% CI)","0.92 (0.88 - 0.96)",NA,"ref","0.78 (0.62 - 0.98)","0.73 (0.63 - 0.84)","0.59 (0.52 - 0.67)","0.51 (0.40 - 0.64)","0.70 (0.50 - 0.98)","0.44 (0.37 - 0.53)","0.42 (0.33 - 0.52)"))

mean=c(NA,**0.92**,NA,**1.00**,**0.78**,**0.73**,**0.59**,**0.51**,**0.70**,**0.44**,**0.42**)

lower=c(NA,**0.88**,NA,**1.00**,**0.62**,**0.63**,**0.52**,**0.40**,**0.50**,**0.37**,**0.33**)

upper=c(NA,**0.96**,NA,**1.00**,**0.98**,**0.84**,**0.67**,**0.64**,**0.98**,**0.53**,**0.52**)

plot.new()

par(xpd=TRUE)

forestplot(tabletext,mean,lower,upper,

graphwidth=unit(**6**,"cm"),

is.summary=c(TRUE,rep(FALSE,**10**)) ,

graph.pos=**4**,

boxsize=**0.2**,zero=**1**,line.margin =**0.1**,#clip=c(**0**,**3.5**),

txt_gp = fpTxtGp(xlab = gpar(cex = **1**) ,ticks = gpar(cex=**1**),

label = gpar(fontfamily = "Times New Roman")),

xticks=c(**0.2**,**0.4**,**0.6**,**0.8**,**1**,**1.2**),

col=fpColors(box=c("black"),line=c( "black")), colgap=unit(**1**,"cm"),cex=**0.9**,

xlog=TRUE ,xlab="HR and 95%CI")

dev.off()

tabletext<-cbind(c("","Per one ideal LS7 metric increase","Change in categories of no. of ideal LS7 metrics"," Persistently poor (0-2)",

" Poor (0-2) → Intermediate (3-4) or High (5-6)"," Intermediate (3-4) → Poor (0-2)"," Persistently intermediate (3-4)"," Intermediate (3-4) → High (5-6)"," High (5-6) → Poor (0-2) "," High (5-6) → Intermediate (3-4)"," Persistently high (5-6)"),

c("n MM/N","3762/52271",NA,"180/1113","79/464","254/1701","1352/13680","238/3692","14/112","498/6243","1147/25266"),

c("IR per 1000 PY","",NA,"18·8","18·7","16·3","10·2","6·6","13·7","8·3","4·6"),

c("HR (95% CI)","0·81 (0·78 - 0·84)",NA,"ref","1·11 (0·85 - 1·45)","0·91 (0·75 - 1·11)","0·66 (0·57 - 0·78)","0·48 (0·39 - 0·58)","0·81 (0·47 - 1·39)","0·58 (0·49 - 0·69)","0·37 (0·31 - 0·43)"))

mean=c(NA,0.81,NA,1.00,1.11,0.91,0.66,0.48,0.81,0.58,0.37)

lower=c(NA,0.78,NA,1.00,0.85,0.75,0.57,0.39,0.47,0.49,0.31)

upper=c(NA,0.84,NA,1.00,1.45,1.11,0.78,0.58,1.39,0.69,0.43)

plot.new()

par(xpd=TRUE)

forestplot(tabletext,mean,lower,upper,

graphwidth=unit(6,"cm"),

is.summary=c(TRUE,rep(FALSE,10)) ,

graph.pos=4,

boxsize=0.2,zero=1,line.margin =0.1,#clip=c(0,3.5),

txt_gp = fpTxtGp(xlab = gpar(cex = 1) ,ticks = gpar(cex=1),

label = gpar(fontfamily = "Times New Roman")) ,

xticks=c(0.2,0.4,0.6,0.8,1,1.2,1.5),

col=fpColors(box=c("black"),line=c( "black")), colgap=unit(1,"cm"),cex=0.9,

xlog=TRUE,xlab="HR and 95% CI")

dev.off()

**************************************************************************

*** Supplementary Table 8 ***

**************************************************************************;

**proc** **format**;

value nbmmgp4p

**1** = '0'

**4** - HIGH = '4'

;

**run**;

**proc** **freq** data=base7 ;format nbmorb nbmmgp4p.;

table nbmorb*multmorb;

**run**;

**proc** **logistic** data=base7;format nbmorb nbmmgp4p.;

class sex ethn_ds edlevr grlump statusx;

model nbmorb(ref='0')=nbid age_c coh sex ethn_ds edlevr grlump statusx/link=glogit rl;

**run**;

**proc** **freq** data=change7 ;format nbmorb nbmmgp4p.;

table nbmorb*multmorb;

**run**;

**proc** **logistic** data=change7;format nbmorb nbmmgp4p.;

class sex ethn_ds edlevr grlump statusx;

model nbmorb(ref='0')=deltnbid nbid age_c coh sex ethn_ds edlevr grlump statusx/link=glogit rl;

**run**;

**************************************************************************

*** Supplementary Table 9 ***

**************************************************************************;

**proc** **format**;

value somcvhgroup

LOW -<**6** = '1 0-5'

**6**-<**10** = '2 6-9'

**10** - HIGH = '3 10-14'

;

**run**;

**proc** **genmod** data=base7;

class somicv;

model multmorb = somicv / dist=poisson offset=lndmm;

lsmeans somicv / exp cl;

format somicv somcvhgroup.;

**run**;

**proc** **phreg** data=base7;format somicv somcvhgroup.;

strata coh;

class somicv(ref='1 0-5') sex ethn_ds edlevr grlump statusx;

model (age_c,age_mm)*multmorb(**0**)=somicv sex ethn_ds edlevr grlump statusx/rl;

**run**;

**proc** **format**;

value $combisomgp

'1 0-52 6-9' = '1 0-5 6-14'

'1 0-53 10-14' = '1 0-5 6-14'

'3 10-141 0-5' = '3 10-14 0-9'

'3 10-142 6-9' = '3 10-14 0-9'

;

**run**;

**proc** **genmod** data=change7;

class combiscorg;

model multmorb = combiscorg / dist=poisson offset=lndmm;

lsmeans combiscorg / exp cl;

format combiscorg $combisomgp.;

**run**;

**proc** **phreg** data=change7;format combiscorg2 $combisomgp.;

strata coh;

class combiscorg(ref='1 0-51 0-5') sex ethn_ds edlevr grlump statusx;

model (xage_c,age_mm)*multmorb(**0**)=combiscorg sex ethn_ds edlevr grlump statusx/rl;

**run**;

**************************************************************************

*** Supplementary Table 11 ***

**************************************************************************;

**proc** **format**;

value metbin

**1** = **0**

**2** = **1**

;

**run**;

**proc** **freq** data=base7;format icv_tabac icv_bmi icv_sport icv_diet icv_blood icv_chol icv_gly metbin.;

table (icv_tabac icv_bmi icv_sport icv_diet icv_blood icv_chol icv_gly )*multmorb;

**run**;

**proc** **phreg** data=base7;format icv_tabac metbin.;

strata coh;

class icv_tabac(ref='0') sex ethn_ds edlevr grlump statusx;

model (age_c,age_mm)*multmorb(**0**)=icv_tabac sex ethn_ds edlevr grlump statusx/rl;

**run**;

**proc** **phreg** data=base7;format icv_bmi metbin.;

strata coh ;

class icv_bmi(ref='0') sex ethn_ds edlevr grlump statusx;

model (age_c,age_mm)*multmorb(**0**)=icv_bmi sex ethn_ds edlevr grlump statusx/rl;

**run**;

**proc** **phreg** data=base7;format icv_sport metbin.;

strata coh ;

class icv_sport(ref='0') sex ethn_ds edlevr grlump statusx;

model (age_c,age_mm)*multmorb(**0**)=icv_sport sex ethn_ds edlevr grlump statusx/rl;

**run**;

**proc** **phreg** data=base7;format icv_diet metbin.;

strata coh ;

class icv_diet(ref='0') sex ethn_ds edlevr grlump statusx;

model (age_c,age_mm)*multmorb(**0**)=icv_diet sex ethn_ds edlevr grlump statusx/rl;

**run**;

**proc** **phreg** data=base7;format icv_blood metbin.;

strata coh ;

class icv_blood(ref='0') sex ethn_ds edlevr grlump statusx;

model (age_c,age_mm)*multmorb(**0**)=icv_blood sex ethn_ds edlevr grlump statusx/rl;

**run**;

**proc** **phreg** data=base7;format icv_chol metbin.;

strata coh ;

class icv_chol(ref='0') sex ethn_ds edlevr grlump statusx;

model (age_c,age_mm)*multmorb(**0**)=icv_chol sex ethn_ds edlevr grlump statusx/rl;

**run**;

**proc** **phreg** data=base7;format icv_gly metbin.;

strata coh ;

class icv_gly(ref='0') sex ethn_ds edlevr grlump statusx;

model (age_c,age_mm)*multmorb(**0**)=icv_gly sex ethn_ds edlevr grlump statusx/rl;

**run**;

**************************************************************************

*** Supplementary Table 12 ***

**************************************************************************;

**proc** **format**;

value combigpbin

**0** = '0'

**1** = '0'

**2** = '1'

**10** = '0'

**11** = '0'

**12** = '1'

**20** = '10'

**21** = '10'

**22** = '11'

;

**run**;

**proc** **freq** data=change7;format chg_: combigpbin.;

table chg_:*multmorb;

**run**;

**proc** **phreg** data=change7;format chg_tabac combigpbin.;

strata coh ;

class chg_tabac(ref='0') sex ethn_ds edlevr grlump statusx;

model (xage_c,age_mm)*multmorb(**0**)=chg_tabac sex ethn_ds edlevr grlump statusx/rl;

**run**;

**proc** **phreg** data=change7;format chg_bmi combigpbin.;

strata coh ;

class chg_bmi(ref='0') sex ethn_ds edlevr grlump statusx;

model (xage_c,age_mm)*multmorb(**0**)=chg_bmi sex ethn_ds edlevr grlump statusx/rl;

**run**;

**proc** **phreg** data=change7;format chg_sport combigpbin.;

strata coh ;

class chg_sport(ref='0') sex ethn_ds edlevr grlump statusx;

model (xage_c,age_mm)*multmorb(**0**)=chg_sport sex ethn_ds edlevr grlump statusx/rl;

**run**;

**proc** **phreg** data=change7;format chg_diet combigpbin.;

strata coh ;

class chg_diet(ref='0') sex ethn_ds edlevr grlump statusx;

model (xage_c,age_mm)*multmorb(**0**)=chg_diet sex ethn_ds edlevr grlump statusx/rl;

**run**;

**proc** **phreg** data=change7;format chg_blood combigpbin.;

strata coh ;

class chg_blood(ref='0') sex ethn_ds edlevr grlump statusx;

model (xage_c,age_mm)*multmorb(**0**)=chg_blood sex ethn_ds edlevr grlump statusx/rl;

**run**;

**proc** **phreg** data=change7;format chg_chol combigpbin.;

strata coh ;

class chg_chol(ref='0') sex ethn_ds edlevr grlump statusx;

model (xage_c,age_mm)*multmorb(**0**)=chg_chol sex ethn_ds edlevr grlump statusx/rl;

**run**;

**proc** **phreg** data=change7;format chg_gly combigpbin.;

strata coh ;

class chg_gly(ref='0') sex ethn_ds edlevr grlump statusx;

model (xage_c,age_mm)*multmorb(**0**)=chg_gly sex ethn_ds edlevr grlump statusx/rl;

**run**;

**************************************************************************

*** Supplementary Table 13 ***

**************************************************************************;

**proc** **logistic** data=attr7 descending ;

class sex ethn_ds edlevr grlump statusx icv_tabac icv_bmi icv_sport icv_diet icv_blood icv_chol icv_gly coh/param=ref;

model cvhph3ok =age_c sex ethn_ds edlevr grlump statusx icv_tabac icv_bmi icv_sport icv_diet icv_blood icv_chol icv_gly coh/rl;

OUTPUT OUT=prob7 PROB=prob;

**run**;

**data** ipw7;set prob7;where cvhph3ok=**1**;

ipw=**1**/prob;**run**;

**proc** **phreg** data=ipw7;

strata coh;weight ipw;

class sex ethn_ds edlevr grlump statusx;

model (xage_c,age_mm)*multmorb(**0**)= deltnbid nbid sex ethn_ds edlevr grlump statusx/rl;

**run**;

**proc** **phreg** data=ipw7;format combi $combigp. ;

strata coh ;weight ipw;

class combi(ref='00') sex ethn_ds edlevr grlump statusx;

model (xage_c,age_mm)*multmorb(**0**)=combi sex ethn_ds edlevr grlump statusx/rl;

**run**;

**proc** **logistic** data=attr8 descending;

class sex ethn_ds edlevr grlump statusx coh/param=ref;

model cvhph5ok =age_c sex ethn_ds edlevr grlump statusx scoret0_smok scoret0_bmi scoret0_pa scoret0_alim scoret0_sleep scoret0_bp scoret0_chol scoret0_diab coh/rl;

OUTPUT OUT=prob8 PROB=prob;

**run**;

**data** ipw8;set prob8;where cvhph5ok=**1**;

ipw=**1**/prob;**run**;

**proc** **phreg** data=ipw8;

strata coh;weight ipw;

class sex ethn_ds edlevr grlump statusx;

model (tage_c,age_mm)*multmorb(**0**)=deltscor scoret0_cvh8 sex ethn_ds edlevr grlump statusx/rl;

hazardratio deltscor/units=**10**;

**run**;

**proc** **phreg** data=ipw8;format combiscore $combiscoregp.;

strata coh;weight ipw;

class sex ethn_ds edlevr grlump statusx;

class combiscore(ref='0 Low0 Low');

model (tage_c,age_mm)*multmorb(**0**)=combiscore sex ethn_ds edlevr grlump statusx/rl;

**run**;

**************************************************************************

*** Supplementary Table 14 ***

**************************************************************************;

**proc** **phreg** data=base7;

strata coh;

model (age_c,age_mm)*mmcomp(**0**)=nbid/eventcode=**1** rl;

**run**;

**proc** **phreg** data=base7;format nbid nbcvhgroup.;

strata coh;

class nbid(ref='0-1');

model (age_c,age_mm)*mmcomp(**0**)=nbid/eventcode=**1** rl;

**run**;

**proc** **phreg** data=change7;

strata coh;

model (xage_c,age_mm)*mmcomp(**0**)=deltnbid nbid/eventcode=**1** rl;

**run**;

**proc** **phreg** data=change7;format combi $combigp. ;

strata coh ;

class combi(ref='00');

model (xage_c,age_mm)*mmcomp(**0**)=combi/eventcode=**1** rl;

**run**;

**************************************************************************

*** Supplementary Table 15 ***

**************************************************************************;

**proc** **phreg** data=base8;

strata coh;

class sex ethn_ds edlevr grlump statusx;

model (age_c,age_mm)*mmcomp(**0**)=scoret0_cvh8 sex ethn_ds edlevr grlump statusx/eventcode=**1** rl;

hazardratio scoret0_cvh8/units=**10**;

**run**;

**proc** **phreg** data=base8;format scoret0_cvh8 cvh8group.;

strata coh;

class scoret0_cvh8(ref='0 Low') sex ethn_ds edlevr grlump statusx;

model (age_c,age_mm)*mmcomp(**0**)=scoret0_cvh8 sex ethn_ds edlevr grlump statusx/eventcode=**1** rl;

**run**;

**proc** **phreg** data=change8;

strata coh;

class sex ethn_ds edlevr grlump statusx;

model (tage_c,age_mm)*mmcomp(**0**)=deltscor scoret0_cvh8 sex ethn_ds edlevr grlump statusx/eventcode=**1** rl;

hazardratio deltscor/units=**10**;

**run**;

**proc** **phreg** data=change8;format combiscore $combiscoregp.;

strata coh;

class sex ethn_ds edlevr grlump statusx;

class combiscore(ref='0 Low0 Low');

model (tage_c,age_mm)*mmcomp(**0**)=combiscore sex ethn_ds edlevr grlump statusx/eventcode=**1** rl;

**run**;

**************************************************************************

*** Supplementary Table 16 ***

**************************************************************************;

*Excluding singly chronic diseases;

**data** b7x;set base7;where nbatcd =**0**;**run**;

**proc** **freq** data=b7x; format nbid nbcvhgroup.;

table nbid*multmorb;

**run**;

**proc** **phreg** data=b7x;

strata coh;

class sex ethn_ds edlevr grlump statusx;

model (age_c,age_mm)*multmorb(**0**)=nbid sex ethn_ds edlevr grlump statusx/rl;

**run**;

**proc** **phreg** data=b7x;format nbid nbcvhgroup.;

strata coh;

class nbid(ref='0-1') sex ethn_ds edlevr grlump statusx;

model (age_c,age_mm)*multmorb(**0**)=nbid sex ethn_ds edlevr grlump statusx/rl;

**run**;

**data** c7x;set change7;where nbatcd=**0** and NOT(nbmorb=**1** and delevt1<=delphase3);

**run**;

**proc** **freq** data=c7x;format combi $combigp.;

table combi*multmorb;

**run**;

**proc** **phreg** data=c7x;

strata coh;

class sex ethn_ds edlevr grlump statusx;

model (age_c,age_mm)*multmorb(**0**)= deltnbid nbid sex ethn_ds edlevr grlump statusx/rl;

**run**;

**proc** **phreg** data=c7x;format combi $combigp. ;

strata coh ;

class combi(ref='00') sex ethn_ds edlevr grlump statusx;

model (age_c,age_mm)*multmorb(**0**)=combi sex ethn_ds edlevr grlump statusx/rl;

**run**;

*Censoring after 15 years;

**data** b7_15;set base7;

multmorb15=multmorb;age_mm15=age_mm;

delmultmorb15=delmultmorb;

if delmultmorb>**15** then do;multmorb15=**0**;delmultmorb15=**15**;age_mm15=age_c+delmultmorb15;end;

**run**;

**proc** **freq** data=b7_15;format nbid nbcvhgroup.;

table nbid*multmorb15;

**run**;

**proc** **phreg** data=b7_15;

strata coh;

class sex ethn_ds edlevr grlump statusx;

model (age_c,age_mm15)*multmorb15(**0**)=nbid sex ethn_ds edlevr grlump statusx/rl;

**run**;

**proc** **phreg** data=b7_15;format nbid nbcvhgroup.;

strata coh;

class nbid(ref='0-1') sex ethn_ds edlevr grlump statusx;

model (age_c,age_mm15)*multmorb15(**0**)=nbid sex ethn_ds edlevr grlump statusx/rl;

**run**;

**data** c7_15;set change7;

multmorb15=multmorb;age_mm15=age_mm;

delmultmorb15=delmultmorb;

if (delmultmorb-delphase3)>**15** then do;multmorb15=**0**;delmultmorb15=**15**+delphase3;age_mm15=age_c+delmultmorb15;end;

**run**;

**proc** **freq** data=c7_15;format combi $combigp.;

table combi*multmorb15;

**run**;

**proc** **phreg** data=c7_15;

strata coh;

class sex ethn_ds edlevr grlump statusx;

model (xage_c,age_mm15)*multmorb15(**0**)= deltnbid nbid sex ethn_ds edlevr grlump statusx/rl;

**run**;

**proc** **phreg** data=c7_15;format combi $combigp. ;

strata coh ;

class combi(ref='00') sex ethn_ds edlevr grlump statusx;

model (xage_c,age_mm15)*multmorb15(**0**)=combi sex ethn_ds edlevr grlump statusx/rl;

**run**;

**************************************************************************

*** Supplementary Table 17 ***

**************************************************************************;

*Excluding singly chronic diseases;

**data** b8x;set base8;where nbatcd =**0**;**run**;

**proc** **freq** data=b8x;format scoret0_cvh8 cvh8group.;

table scoret0_cvh8*multmorb;

**run**;

**proc** **phreg** data=b8x;

strata coh;

class sex ethn_ds edlevr grlump statusx;

model (age_c,age_mm)*multmorb(**0**)=scoret0_cvh8 sex ethn_ds edlevr grlump statusx/rl;

hazardratio scoret0_cvh8/units=**10**;

**run**;

**proc** **phreg** data=b8x;format scoret0_cvh8 cvh8group.;

strata coh;

class scoret0_cvh8(ref='0 Low') sex ethn_ds edlevr grlump statusx;

model (age_c,age_mm)*multmorb(**0**)=scoret0_cvh8 sex ethn_ds edlevr grlump statusx/rl;

**run**;

**data** c8x;set change8;where nbatcd=**0** and NOT(nbmorb=**1** and delevt1<=delphase5);

**run**;

**proc** **freq** data=c8x;format combiscore $combiscoregp.;

table combiscore*multmorb;**run**;

**proc** **phreg** data=c8x;

strata coh;

class sex ethn_ds edlevr grlump statusx;

model (tage_c,age_mm)*multmorb(**0**)=deltscor scoret0_cvh8 sex ethn_ds edlevr grlump statusx/rl;

hazardratio deltscor/units=**10**;

**run**;

**proc** **phreg** data=c8x;format combiscore $combiscoregp.;

strata coh;

class sex ethn_ds edlevr grlump statusx;

class combiscore(ref='0 Low0 Low');

model (tage_c,age_mm)*multmorb(**0**)=combiscore sex ethn_ds edlevr grlump statusx/rl;

**run**;

*Censoring after 15 years;

**data** b8_15;set base8;

multmorb15=multmorb;age_mm15=age_mm;

delmultmorb15=delmultmorb;

if delmultmorb>**15** then do;multmorb15=**0**;delmultmorb15=**15**;age_mm15=age_c+delmultmorb15;end;

**run**;

**proc** **freq** data=b8_15;format scoret0_cvh8 cvh8group.;

table scoret0_cvh8*multmorb15;

**run**;

**proc** **phreg** data=b8_15;

strata coh;

class sex ethn_ds edlevr grlump statusx;

model (age_c,age_mm15)*multmorb15(**0**)=scoret0_cvh8 sex ethn_ds edlevr grlump statusx/rl;

hazardratio scoret0_cvh8/units=**10**;

**run**;

**proc** **phreg** data=b8_15;format scoret0_cvh8 cvh8group.;

strata coh;

class scoret0_cvh8(ref='0 Low')sex ethn_ds edlevr grlump statusx;

model (age_c,age_mm15)*multmorb15(**0**)=scoret0_cvh8 sex ethn_ds edlevr grlump statusx/rl;

**run**;

**data** c8_15;set change8;

multmorb15=multmorb;age_mm15=age_mm;

delmultmorb15=delmultmorb;

if (delmultmorb-delphase3)>**15** then do;multmorb15=**0**;delmultmorb15=**15**+delphase3;age_mm15=age_c+delmultmorb15;end;

**run**;

**proc** **freq** data=c8_15;format combiscore $combiscoregp.;

table combiscore*multmorb15;

**run**;

**proc** **phreg** data=c8_15;

strata coh;

class sex ethn_ds edlevr grlump statusx;

model (tage_c,age_mm15)*multmorb15(**0**)= deltscor scoret0_cvh8 sex ethn_ds edlevr grlump statusx/rl;

hazardratio deltscor/units=**10**;

**run**;

**proc** **phreg** data=c8_15;format combiscore $combiscoregp.;

strata coh ;

class sex ethn_ds edlevr grlump statusx combiscore(ref='0 Low0 Low');

model (tage_c,age_mm15)*multmorb15(**0**)=combiscore sex ethn_ds edlevr grlump statusx/rl;

**run**;

**************************************************************************

*** Supplementary Table 18 ***

**************************************************************************;

**data** b7xcvd;set base7;if multmorb=**1** and (chd=**1** or stroke=**1** or hfailure=**1**) then delete;**run**;*8172;

**proc** **freq** data=b7xcvd; format nbid nbcvhgroup.;

table nbid*multmorb;

**run**;

**proc** **phreg** data=b7xcvd;

strata coh;

class sex ethn_ds edlevr grlump statusx;

model (age_c,age_mm)*multmorb(**0**)=nbid sex ethn_ds edlevr grlump statusx/rl;

**run**;

**proc** **phreg** data=b7xcvd;format nbid nbcvhgroup.;

strata coh;

class nbid(ref='0-1') sex ethn_ds edlevr grlump statusx;

model (age_c,age_mm)*multmorb(**0**)=nbid sex ethn_ds edlevr grlump statusx/rl;

**run**;

**data** c7xcvd;set change7;if multmorb=**1** and (chd=**1** or stroke=**1** or hfailure=**1**)then delete;**run**;

**proc** **freq** data=c7xcvd;format combi $combigp.;

table combi*multmorb;

**run**;

**proc** **phreg** data=c7xcvd;

strata coh;

class sex ethn_ds edlevr grlump statusx;

model (xage_c,age_mm)*multmorb(**0**)= deltnbid nbid sex ethn_ds edlevr grlump statusx/rl;

**run**;

**proc** **phreg** data=c7xcvd;format combi $combigp. ;

strata coh ;

class combi(ref='00') sex ethn_ds edlevr grlump statusx;

model (xage_c,age_mm)*multmorb(**0**)=combi sex ethn_ds edlevr grlump statusx/rl;

**run**;

**************************************************************************

*** Supplementary Table 19 ***

**************************************************************************;

**data** b8xcvd;set base8;if multmorb=**1** and (chd=**1** or stroke=**1** or hfailure=**1**) then delete;**run**;

**proc** **freq** data=b8xcvd;format scoret0_cvh8 cvh8group.;

table scoret0_cvh8*multmorb;

**run**;

**proc** **phreg** data=b8xcvd;

strata coh;

class sex ethn_ds edlevr grlump statusx;

model (age_c,age_mm)*multmorb(**0**)=scoret0_cvh8 sex ethn_ds edlevr grlump statusx/rl;

hazardratio scoret0_cvh8/units=**10**;

**run**;

**proc** **phreg** data=b8xcvd;format scoret0_cvh8 cvh8group.;

strata coh;

class scoret0_cvh8(ref='0 Low') sex ethn_ds edlevr grlump statusx;

model (age_c,age_mm)*multmorb(**0**)=scoret0_cvh8 sex ethn_ds edlevr grlump statusx/rl;

**run**;

**data** c8xchd;set change8;if multmorb=**1** and (chd=**1** or stroke=**1** or hfailure=**1**) then delete;**run**;

**proc** **freq** data=c8xchd;format combiscore $combiscoregp.;

table combiscore*multmorb;**run**;

**proc** **phreg** data=c8xchd;

strata coh;

class sex ethn_ds edlevr grlump statusx;

model (tage_c,age_mm)*multmorb(**0**)=deltscor scoret0_cvh8 sex ethn_ds edlevr grlump statusx/rl;

hazardratio deltscor/units=**10**;

**run**;

**proc** **phreg** data=c8xchd;format combiscore $combiscoregp.;

strata coh;

class sex ethn_ds edlevr grlump statusx;

class combiscore(ref='0 Low0 Low');

model (tage_c,age_mm)*multmorb(**0**)=combiscore sex ethn_ds edlevr grlump statusx/rl;

**run**;

**************************************************************************

*** Supplementary Table 20 ***

**************************************************************************;

**proc** **freq** data=base7;format nbid nbcvhgroup.;where cmiss(hafamily,strfamily,angfamily,diabfamily,fhist_kc,XADTOWNS,alc)=**0**;

table nbid*multmorb;

**run**;

**proc** **phreg** data= base7;format nbid nbcvhgroup.;

strata coh;

class nbid(ref='0-1') sex ethn_ds edlevr grlump statusx hafamily(ref='2') strfamily(ref='2') angfamily(ref='2') diabfamily(ref='2') alc(ref='0');

model (age_c,age_mm)*multmorb(**0**)=nbid sex ethn_ds edlevr grlump statusx hafamily strfamily angfamily diabfamily fhist_kc XADTOWNS alc/rl;

**run**;

**proc** **phreg** data= base7;

strata coh;

class sex ethn_ds edlevr grlump statusx hafamily(ref='2') strfamily(ref='2') angfamily(ref='2') diabfamily(ref='2') alc(ref='0');

model (age_c,age_mm)*multmorb(**0**)=nbid sex ethn_ds edlevr grlump statusx hafamily strfamily angfamily diabfamily fhist_kc XADTOWNS alc/rl;

**run**;

**proc** **freq** data=change7;format combi $combigp.;where cmiss(hafamily,strfamily,angfamily,diabfamily,fhist_kc,XADTOWNS,alc)=**0**;

table combi*multmorb;

**run**;

**proc** **phreg** data=change7;format combi $combigp. ;

strata coh ;

class combi(ref='00') sex ethn_ds edlevr grlump statusx hafamily(ref='2') strfamily(ref='2') angfamily(ref='2') diabfamily(ref='2') alc(ref='0');

model (xage_c,age_mm)*multmorb(**0**)=combi sex ethn_ds edlevr grlump statusx hafamily strfamily angfamily diabfamily fhist_kc XADTOWNS alc/rl;

**run**;

**proc** **phreg** data= change7;

strata coh;

class sex ethn_ds edlevr grlump statusx hafamily(ref='2') strfamily(ref='2') angfamily(ref='2') diabfamily(ref='2') alc(ref='0');

model (xage_c,age_mm)*multmorb(**0**)= deltnbid nbid sex ethn_ds edlevr grlump statusx hafamily strfamily angfamily diabfamily fhist_kc XADTOWNS alc/rl;

**run**;

**************************************************************************

*** Supplementary Table 21 ***

**************************************************************************;

**proc** **freq** data=base8;format scoret0_cvh8 cvh8group.;where cmiss(hafamily,strfamily,angfamily,diabfamily,fhist_kc,XADTOWNS,alc)=**0**;

table scoret0_cvh8*multmorb;

**run**;

**proc** **phreg** data= base8;format scoret0_cvh8 cvh8group.;

strata coh;

class sex ethn_ds edlevr grlump statusx hafamily(ref='2') strfamily(ref='2') angfamily(ref='2') diabfamily(ref='2') alc(ref='0') scoret0_cvh8(ref='0 Low');

model (age_c,age_mm)*multmorb(**0**)=scoret0_cvh8 sex ethn_ds edlevr grlump statusx hafamily strfamily angfamily diabfamily fhist_kc XADTOWNS alc/rl;

hazardratio scoret0_cvh8/units=**10**;

**run**;

**proc** **phreg** data= base8;

strata coh;

class sex ethn_ds edlevr grlump statusx hafamily(ref='2') strfamily(ref='2') angfamily(ref='2') diabfamily(ref='2') alc(ref='0');

model (age_c,age_mm)*multmorb(**0**)=scoret0_cvh8 sex ethn_ds edlevr grlump statusx hafamily strfamily angfamily diabfamily fhist_kc XADTOWNS alc/rl;

hazardratio scoret0_cvh8/units=**10**;

**run**;

**proc** **freq** data=change8;format combiscore $combiscoregp.;where cmiss(hafamily,strfamily,angfamily,diabfamily,fhist_kc,XADTOWNS,alc)=**0**;

table combiscore*multmorb;

**run**;

**proc** **phreg** data= change8;format combiscore $combiscoregp.;

strata coh;

class sex ethn_ds edlevr grlump statusx hafamily(ref='2') strfamily(ref='2') angfamily(ref='2') diabfamily(ref='2') alc(ref='0');

class combiscore(ref='0 Low0 Low');

model (tage_c,age_mm)*multmorb(**0**)=combiscore sex ethn_ds edlevr grlump statusx hafamily strfamily angfamily diabfamily fhist_kc XADTOWNS alc/rl;

**run**;

**proc** **phreg** data= change8;

strata coh;

class sex ethn_ds edlevr grlump statusx hafamily(ref='2') strfamily(ref='2') angfamily(ref='2') diabfamily(ref='2') alc(ref='0');

model (tage_c,age_mm)*multmorb(**0**)=deltscor scoret0_cvh8 sex ethn_ds edlevr grlump statusx hafamily strfamily angfamily diabfamily fhist_kc XADTOWNS alc/rl;

hazardratio deltscor/units=**10**;

**run**;

Finnish Public Sector Study / Statistical code (SAS):

********************************************************************;

*** Table 1 ***;

********************************************************************;

**proc** **freq** data=base;

tables sex educ occup mstatus CVH_ideal;

**proc** **means** data=base;

var age CVH_ideal;

**run**;

********************************************************************;

*** Table 2, Supplementary Table 10 ***;

********************************************************************;

**proc** **format**;

value lkm **0**-**1**='0-1';

**proc** **freq** data=base;

tables (CVH_ideal CVH_ideal_s)*status_mm / nopercent nocol norow;

format CVH_ideal CVH_ideal_s lkm.;

**run**;

* Incidence rate per 1000 py (lnfyears=log(fyears/1000) *;

**proc** **genmod** data=base;

model status_mm = / dist=poisson offset=lnfyears;

**proc** **genmod** data=base;

class CVH_ideal(ref='0-1');

model status_mm = CVH_ideal / dist=poisson offset=lnfyears;

lsmeans CVH_ideal / exp cl;

format CVH_ideal lkm.;

**run**;

* Hazard ratios *;

**proc** **phreg** data=base;

class occup educ CVH_ideal(ref='0-1');

model (age,age_mm)*status_mm(**0**)= sex educ occup mstatus CVH_ideal / rl;

strata byear (**1940** to **1990** by **10**);

format CVH_ideal lkm.;

**proc** **phreg** data=base;

class occup educ;

model (age,age_mm)*status_mm(**0**)= sex educ occup mstatus CVH_ideal / rl;

strata byear (**1940** to **1990** by **10**);

**run**;

/* Test for the linearity assumption: difference in log likelihood ratio

between model without and with a quadratic term on CVH exposure*/

**proc** **phreg** data=base;

class occup educ;

model (age,age_mm)*status_mm(**0**)= sex educ occup mstatus CVH_ideal / rl;

strata byear (**1940** to **1990** by **10**);

**proc** **phreg** data=base;

class occup educ;

model (age,age_mm)*status_mm(**0**)= sex educ occup mstatus CVH_ideal CVH_ideal*CVH_ideal / rl;

strata byear (**1940** to **1990** by **10**);

**run**;

********************************************************************;

*** Supplementary Table 8 ***;

********************************************************************;

**proc** **freq** data=base;

tables mm; *1-4*;

**proc** **logistic** data=base;

class occup educ;

model mm(ref='1') = age byear sex educ occup mstatus CVH_ideal / link=glogit;

**run**;

**proc** **freq** data=change;

tables mm;

**proc** **logistic** data=change;

class occup educ;

model mm(ref='1') = age2 byear sex educ occup mstatus CVH_ideal CVH_ideal_change / link=glogit;

**run**;

********************************************************************;

*** Figure 1B ***;

********************************************************************;

**data** apu1; set change;

time=round(fyears_mm,**0.1**);

group=CVH_ideal_changeC;

**proc** **phreg** data=apu1;

model time*status_mm(**0**) = ;

strata group;

baseline out=apu2 survival=survival;

**run**;

**data** apu3;

set apu2;

by group;

retain prehazard **0**;

hazard=**1**-survival;

if (hazard>**.** and hazard NE prehazard) or first.group;

prehazard=hazard;

keep group time hazard;

**data** apu4;

set apu3;

retain prehazard pregroup **0**;

if group=pregroup then do;

cumhazard=prehazard; output; end;

cumhazard=hazard; output;

pregroup=group;

prehazard=hazard;

keep group time cumhazard;

**data** apu4;

set apu4;

cumhazard=**100***cumhazard; * % *;

**run**;

**proc** **print** data=apu4; * => Excel figure *;

**run**;

* Number at risk *;

**data** n1;

set apu1;

y0n=**0**; y5n=**0**; y10n=**0**;

if time>=**0** then y0n=**1**;

if time>=**5** then y5n=**1**;

if time>=**10** then y10n=**1**;

**proc** **means** data=n1 sum;

var y0n--y10n;

class group;

**run**;

********************************************************************;

*** Figure 2B, Supplementary Table 10 ***;

********************************************************************;

**proc** **freq** data=change;

tables (CVH_ideal_changeC CVH_ideal_change_sC)*status_mm / nopercent nocol norow;

**run**;

* Incidence rate per 1000 py *;

**proc** **genmod** data=change;

model status_mm = / dist=poisson offset=lnfyears2; *lnfyears2=log(fyears2/1000);

**run**;

**proc** **genmod** data=change;

class CVH_ideal_changeC(ref='00');

model status_mm = CVH_ideal_changeC / dist=poisson offset=lnfyears2;

lsmeans CVH_ideal_changeC / exp cl;

**run**;

* Hazard ratios *;

**proc** **phreg** data=change;

class occup educ;

model (age2,age_mm)*status_mm(**0**)= sex educ occup mstatus CVH_ideal CVH_ideal_change / rl;

strata byear (**1940** to **1990** by **10**);

**run**;

**proc** **phreg** data=change;

class occup educ CVH_ideal_changeC(ref='00');

model (age2,age_mm)*status_mm(**0**)= sex educ occup mstatus CVH_ideal_changeC / rl;

strata byear (**1940** to **1990** by **10**);

**run**;

********************************************************************;

*** Supplementary Table 11 ***;

********************************************************************;

**proc** **freq** data=base;

tables (smoke met bmi hypert stat diab sdurat)*status_mm / nopercent nocol norow;

**run**;

**proc** **phreg** data=base;

class occup educ;

model (age,age_mm)*status_mm(**0**)= sex educ occup mstatus smoke / rl;

strata byear (**1940** to **1990** by **10**);

**run**;

********************************************************************;

*** Supplementary Table 12 ***;

********************************************************************;

**proc** **freq** data=change;

tables (smoke_change met_change bmi_change hypert_change diab_change stat_change sdurat_change)*status_mm / nopercent nocol norow;

**run**;

**proc** **phreg** data=change;

class occup educ smoke_change(ref='0');

model (age2,age_mm)*status_mm(**0**)= sex educ occup mstatus smoke_change / rl;

strata byear (**1940** to **1990** by **10**);

**run**;

References

1. Bergenstal RM, Beck RW, Close KL, et al. Glucose Management Indicator (GMI): A New Term for Estimating A1C From Continuous Glucose Monitoring. *Diabetes Care* 2018; **41**: 2275–80.

2. Leskinen T, Stenholm S, Heinonen OJ, et al. Change in physical activity and accumulation of cardiometabolic risk factors. *Prev Med* 2018; **112**: 31–7.

3. Fine JP, Gray RJ. A Proportional Hazards Model for the Subdistribution of a Competing Risk. *Journal of the American Statistical Association* 1999; **94**: 496–509.
